# Ecological comparison of native (*Apis mellifera mellifera*) and hybrid (Buckfast) honeybee drones in southwestern Sweden indicates local adaptation

**DOI:** 10.1371/journal.pone.0308831

**Published:** 2024-08-13

**Authors:** Finja Schaumann, Niclas Norrström, Mats Niklasson, Sonja Leidenberger

**Affiliations:** 1 Department of Biology and Bioinformatics, School of Bioscience, University of Skövde, Skövde, Sweden; 2 Nordens Ark Foundation, Åby Säteri, Hunnebostrand, Sweden; 3 Southern Swedish Forest Research Centre, Swedish University of Agricultural Sciences, Lomma, Sweden; University of Alberta, CANADA

## Abstract

Honeybee drones’ only known task is to mate with a virgin queen. Apart from their mating behaviour, their ecology has been little studied, especially in comparison to honeybee females. Previous knowledge is primarily based on short-term direct observations at single experimental hives, rarely, if ever, addressing the effect of drones’ genetic origin. Here, Radio Frequency Identification Technology was utilised to gather drone and worker bee lifetime data of *Apis mellifera mellifera* and *Apis mellifera x* (hybrid Buckfast) colonies over one mating season (spring and summer) with the ultimate goal to investigate differences at subspecies level. This technique enabled continuous monitoring of tagged bees at the hive entrance and recording of individuals’ movement directions. The results confirmed that spring-born drones survive longer than summer-born drones and that they generally live longer than worker bees. Drones’ peak activity occurred in the afternoon while worker bees showed more even activity levels throughout the day. Earlier orientation flights than usually reported for drones were observed. In summer, mating flights were practiced before reaching sexual maturity (at 12 days of age). Differences were found between *Apis m*. *mellifera* and Buckfast drones, where *Apis m*. *mellifera* showed later drone production in spring, but significantly earlier first activities outside the hive in summer and a later peak in diurnal activity. Additionally, *Apis m*. *mellifera* flew more in higher light intensities and windy conditions and performed significantly longer flights than Buckfast drones. The observed differences in drone ecology indicate the existence of a local adaptation of the native subspecies *Apis m*. *mellifera* to environmental conditions in southwestern Sweden.

## Introduction

The western honeybee (*Apis mellifera*, Linnaeus 1758) lives eusocially in large colonies which consist of one fertile queen, many worker bees (females, usually non-fertile), and males, also known as drones. The ecology, behaviour, and genetics of *Apis mellifera* females has been extensively studied with various research objectives, e.g., [1–5], but drones are often neglected as they are not involved in brood or honey production. Their main task is to mate with a virgin queen, and it is assumed that drones also play a role in the thermoregulation inside the hive [6, 7]. Most focus has been set on drones’ mating behaviour at the so called ‘drone congregation areas’ (DCAs), where drones gather and wait for virgin queens to mate with and die thereafter, e.g., [8–13].

Knowledge about drone ecology is especially of interest for beekeeping and breeding programs as the reproductive cycle of *Apis mellifera* colonies is protandrous, e.g., the rearing of drones precedes the production of virgin queens, and the highest number of mature drones coincides with the time for swarming [14]. Drones stay within the hive before their first short orientation flights at an age of about eight days. As soon as they reach maturity at an age of about 12 days, they perform longer mating flights, usually between 2 and 4.30 PM in the afternoon [15–19]. [20] found that older drones were able to return to their colony from a greater distance than younger drones, possibly due to a learning process enabling them to expand their homing range, but drone drift, where drones fly into wrong hives, has been reported to occur at all ages [21, 22]. Reported drone lifespans vary broadly, from 21 days [23] to 90 days [24], most likely depending on seasonal, geographical, and methodological differences of the studies. Drones only live during a single mating season as they get evicted from the hive by worker bees when the virgin queen to mate with is absent [25].

Most previous drone studies have used direct observations or video recordings at a single experimental hive, hence collecting data from a limited number of drones during a short period of time [15, 16, 24, 26–29]. Two recent studies on flight activity over a whole mating season, from France and Argentina [19, 23], also reported a peak in activity of drones in the afternoon. [30] observed in-nest movement behaviour of drones during a whole mating season in southern Germany and found that high activity within the hive relates to the times drones fly actively outside of the hive. However, no study has yet been reported from Scandinavia, where the bee season is characterized by short summers and long winters [31].

The native honeybee of Northern Europe, *Apis mellifera mellifera* (*Mel*) (Linnaeus, 1758), is adapted to the climatic conditions present in the Nordic-Baltic region [32]. The introduction of other *Apis mellifera* subspecies, particularly the hybrid *Apis mellifera x* (so-called Buckfast) has outcompeted local *Mel* populations which reduces their adaptive potential and resilience. Thus, maintaining the genetic integrity of native honeybee populations is crucial, especially in the face of climate change and the challenges it poses [33]. *Apis mellifera* subspecies show beneficial behavioural adaptations when kept in an environment similar to their genetic origin [31, 34, 35]. However, studies comparing the biology of different subspecies within the same geographical area are rare. In Sweden and Norway large populations of *Mel* exist within isolated mating areas [33], supplying pure colonies for sufficient conservation work. Out of 160,000–170,000 honeybee colonies in Sweden [36], only 1,000–1,200 colonies can be attributed to *Mel* [37] while Buckfast (*Buck*) colonies are most commonly used in beekeeping in Sweden with various breeding stations in the entire country [38].

The objective of this study was to analyse and compare drones’ lifespan and flight activity in *Mel* and *Buck* colonies during one mating season with the use of the Radio Frequency Identification Technology (RFID). The study analysed in one apiary i) the lifespan of drones; ii) the age at first activity outside the hive; iii) the daily activity pattern; iv) the influence of weather on activity; v) if the length of flight increases with age; and vi) if drones and worker bees show differences in i—v. As the focus was set on using the RFID technology to collect this data on drones, the comparison to worker bees was used as a control analysis.

## Materials & methods

### Study sites and bee colonies

**Fig 1:**
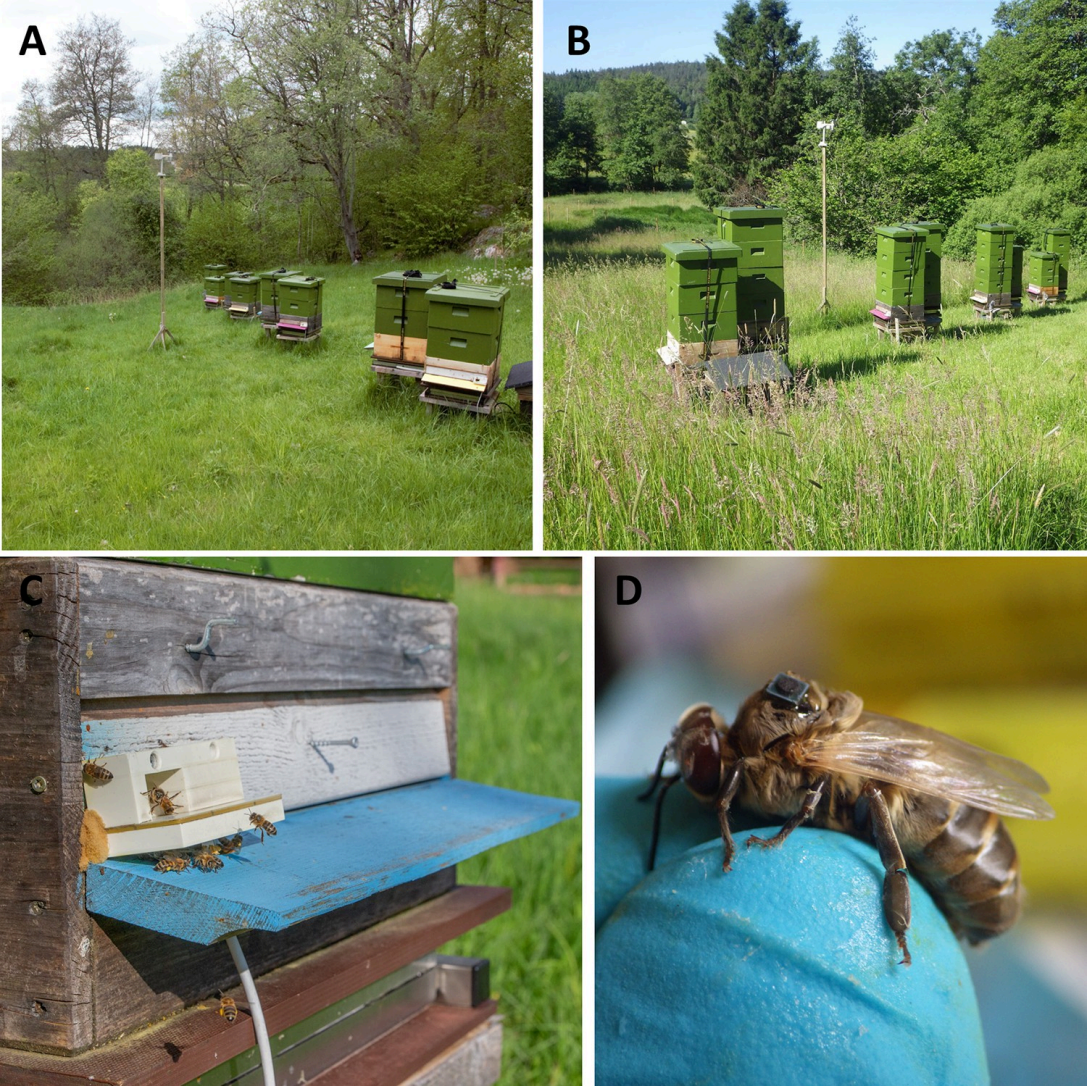
Experimental setup of the apiary in Uddevalla. A Radio Frequency Identification Technology system was installed at each colony to track tagged bees’ activities. Bees were tagged with passive ultra-high frequency tags for the experiment in the first four hives from the left in A) and in the first four hives from the right in B). Each hive entrance consisted of an open tunnel system with integrated antennas recording the direction of movement of a tagged bee, as shown in C). An example of a tagged drone is shown in D). The weather station was placed in the middle of the apiary. Picture A) was taken on 23^rd^ of May 2022, B) on 24^th^ of June 2022, C) on 26^th^ of May 2022 and D) on 18^h^ of May 2022.

An apiary in Uddevalla, southwestern Sweden, was used for the study (58.295922°N, 11.992339°E, 76 m.a.s.l.). The study site was formerly used for the research project *Supporting Nordic brown bees–a unique resource for our ecosystem services* from 2019–2022 [31, 39]. On the 2^nd^ of May 2022, eight beehives were placed in one line (Fig 1) with an alternating order of *Apis mellifera mellifera* (*Mel*), n = 4, and *Buckfast* (*Buck*), n = 4, but only drones and worker bees from four colonies (n = 2 *Mel*, n = 2 *Buck*) were tagged and observed under full tracking purposes (S1 Table). The colonies were formed with queens originating from different breeders in Sweden to present a high genetic variability.

### Monitoring techniques

#### Lifespan and flight activity

To record the lifespan and activity time of drones and worker bees, Radio Frequency Identification (RFID) technology by Microsensys GmbH, Germany, was used. Two colonies from each subspecies (n = 2) were equipped with the antennae system 1 (AEB-03.C2D EU, Microsensys GmbH, Germany) that were able to register the direction of movement via two internal antennas, namely ‘arrival’, ‘departure’ and ‘unknown’ direction. An ‘arrival’ was detected if a tagged bee first crossed the outer and then the inner antenna. The other way around led to a ‘departure’ registration. An ‘unknown’ registration occurred if a tag got registered by one antenna only.

Two additional colonies from each subspecies (n = 2) were equipped with the antennae system 2 (AEW-01.E1D, Microsensys GmbH, Germany) that only captured ‘unknown’ registrations without direction. Both systems were integrated inside an open tunnel system (reader) and externally fixated at every hives’ entrance (Fig 1C). The remaining entrance to the hive was closed to prevent bees, particularly worker bees, from avoiding the reader. All readers were connected to an iID controller (iID®controller CCO, Microsensys GmbH, Germany) that registered the timestamp (in Coordinated Universal Time: UTC), antenna ID, unique tag ID and direction.

The experiment started on the 2^nd^ of May 2022 and all hives were regularly checked for the presence of drone cells, freshly hatched drones and adult drones. Only *Buck* and *Mel* drones as well as worker bees from the four hives connected to system 1 were tagged in two separate cohorts (spring: 18^th^ of May and summer: 14^th^ of June, Fig 1). System 2 was used to detect drifting of drones only. Both cohorts were followed to the 31^st^ of August 2022. We monitored an extra 19 days after the very last registration (12^th^ of August) in order not to miss late registrations.

For each season and in each hive, the goal was to tag 30 drones and 20 worker bees on their thorax with passive ultra-high frequency tags (1.6 x 1.6 x 0.4 mm^3^, 860–868 MHz, TAG mic3®Q1.6, Microsensys GmbH, Germany) by using superglue (Superlim from Biltema Sweden AB containing cyanoacrylate) (Fig 1D). After tagging a bee, the tag was scanned with the IID PENsolid UHFcc (Microsensys GmbH, Germany) to obtain the unique tag ID, which allowed the direct assignment of the bees to their natal colony. In May, only *Buck* drones could be tagged due to the comparably slower drone development of *Mel* drones. In total, 163 drones and 158 worker bees were tagged (S2 Table). For accurate calculations, only freshly hatched drones and worker bees with an apparent maximum age of 72 hours were randomly chosen to be tagged. Freshly hatched *Buck* and *Mel* drones and worker bees have more hair, a greyer appearance, cannot fly, and present a different movement pattern compared to older bees (e.g. [40, 41]).

#### Weather data

In the apiary, temperature (°C), humidity (%), rain (mm) and wind speed (km/h) were recorded every five minutes with an ApiWeather-RF6 weather station (Wolf-Waagen GmbH & Co, Germany). In addition, hourly light intensity data (photo active radiation (PAR), W/m^2^) was downloaded from the mesoscale Strång model (strang.smhi.se) offered by the Swedish Meteorological and Hydrological Institute (S1A–S1E Fig, S3 Table).

#### Categorisation of RFID registrations

The timestamps of all RFID registrations were converted from UTC to Central European time (CET). Based on this, all RFID registrations were classified into four time intervals, adapted from [42]. Registrations from 6–11 AM were defined as MORNING registrations, 11 AM—4 PM as MIDDAY registrations, 4–9 PM as EVENING registrations and 9 PM—6 AM as NIGHT registrations. The Swedish NIGHT time interval included daylight, especially towards midsummer (21^st^ of June), where the sun rose at 4.06 AM and set at 10.21 PM.

A departure and its subsequent arrival registration were counted as a flight. The time at departure was taken for the categorisation into the four time intervals. Flight duration was determined as the timespan between these two registrations. Each flight was classified into one behavioural category, based on [19] (Table 1).

**Table 1:** Behaviour interpretation in terms of the length of flight categories.

Category [min]	Behaviours
< 3	defecate, orientate
3–10	orientation flight, walking around the hive entrance
10–30	short mating flight
30–60	long mating flight
> 60	very long mating flight

These behavioural interpretations only apply to drones. Worker bees’ flight durations were classified into the same categories for comparison only. Each worker’s flight was also classified into the different times of the day.

### Data analysis

All statistical analyses were run in R v. 4.2.3 [43]. Visualisation was done with *ggplot2* (v. 3.4.1) together with colour palettes from the *RColorBrewer* package (v. 1.1–3). Data manipulation was conducted with *tidyverse* packages, such as *dplyr* (v. 1.1.0). Descriptive statistics were performed with the *stats* package (v. 4.2.3). Significance threshold was set to 0.05 for all analyses.

#### Model selection

To analyse factors influencing the hourly number of registrations (*counts*), e.g., age, time interval, and weather, a generalized linear mixed model (GLMM) was developed by using the package *glmmTMB* (v.1.1.6), similar to [44]. The environmental variables were centred, and the negative binomial response distribution ‘nbinom1’ was chosen to allow for overdispersion [45]. Random effects of the colonies and seasons were included to account for the fact that the colonies had different sizes and were measured repeatedly in addition to the different number of tagged bees during both seasons. The variable hour was included as a zero-inflation term to account for possible missed registrations during different levels of activity. It was assumed that the higher the activity at the entrances, the greater the probability that the antennae will miss to register all RFID tags. Registrations from the first two days after each tagging event were removed from the dataset to account for registrations representing possible evictions from the hive rather than an activity relevant for this study. Registrations at each hour (0–23) from system 1 and 2 during the experiment were counted and summed to an hourly count for each hive. Due to many registrations of unknown direction all movement directions were counted. If there was no registration at a certain hour, the hourly count value was set to zero. All drone *Mel* counts during the spring were set to ‘NA’ due to missing data. The variance inflation factor (VIF) of each predictor variable was calculated in a non-interaction model, while in the actual model the weather covariates were tested in interaction with subspecies to determine differences. A backward stepwise model selection was performed by calculating and comparing the Akaike Information Criterion (AIC) of different variations of the models and the validity of each model was assessed by the visualisation of the residuals with the *DHARMa* package (v. 0.4.6). Post analysis of the final model was done with the *parameters* (v. 0.20.2) and *effects* (v. 4.2–2) packages.

#### Lifespan

The lifespan was calculated as the difference in days between the tagging date and the date of the last registration (taking place at own or foreign hive). The Kaplan-Meier method was used to estimate survival times and probabilities (*survival* v. 3.5–5). Survival curves were plotted by using the *survminer* package (v. 0.4.9) and compared with the *survdiff* function (log rank test) from the *survival* package. Furthermore, the effect of age on the hourly number of registrations was evaluated in the zero-inflated GLMM in interaction with subspecies.

#### Age at first activity

The age at first activity was determined by the time difference between the tagging date and the date of first registration. Due to a non-parametric data distribution, the Mann-Whitney-U-Test was used to test for differences.

#### Diurnal activity

Peak activity was determined by the highest number of registrations per time interval in relation to the total number of registrations in order to enable a direct comparison between the groups. The effects of subspecies (∈ (*Buck*, *Mel*)) and individual type (∈ (drones, worker bees)), respectively, and time interval on the number of registrations were analysed with a factorial ANOVA. Due to unbalanced designs, the Type II sums of squares approach was used [46] and the count variable was log-transformed to normalize the data. Tukey’s post hoc analysis was used to evaluate differences between the groups. Furthermore, the effect of the time interval on the hourly number of registrations was tested in the zero-inflated GLMM without any interaction as differences were analysed within the ANOVA.

#### Weather

The average weather conditions for each hour, along with hourly light intensity data, were calculated and matched to the corresponding RFID registrations. Spearman correlations between the number of registrations and weather parameters (temperature, light intensity, wind speed and rain sum) were calculated for each day. The median values were used for interpretation as in [47]. Additionally, the effect of the weather parameters on the hourly number of registrations was evaluated in the zero-inflated GLMM in interaction with subspecies.

#### Flights

The relative number of flights within each category was calculated and visualized. A Spearman correlation analysis between the age of the bees (days) and flight duration (min) was performed and differences between distributions were tested with the Mann-Whitney-U-Test.

#### Ethical statement

The study does not require an ethics statement since experiments involving invertebrates have no legislation restrictions in Sweden.

## Results

RFID registrations of drone activity were recorded from the 18^th^ of May 2022 until the 12^th^ of August 2022. Thereafter, no registrations were observed until the end of the experiment (31^st^ of August). Out of the 163 tagged drones, 133 (81.6%) were registered at least one time. 19 drones only got registered once. Drones’ median number of registrations was 23 (IQR = 245) with a maximum of 490 registrations (a *Buck* drone tagged in spring). The number of registrations did not differ between *Buck* and *Mel* drones. In total, 65 drones (48.8%) were registered in non-natal colonies which account for 33% of all drone registrations.

### Model selection

The collinearities between the weather parameters (S4 Table) did not seem to affect the confidence in the GLMM as for all variables the VIF were less than four [45].

The model excluding the interaction of subspecies and rain but including the interaction between temperature and light intensity showed the lowest AIC value (S5 Table) together with an acceptable fit to the data (S2 Fig) and was chosen as the final model for drones:

counts∼Age+Temperature+Light+Wind+Timeinterval+Subspecies+Subspecies:Age+Subspecies:Temperature+Subspecies:Light+Subspecies:Wind+Temperature:Light−1+(1|Colony)+(1|Season).

**Fig 2:**
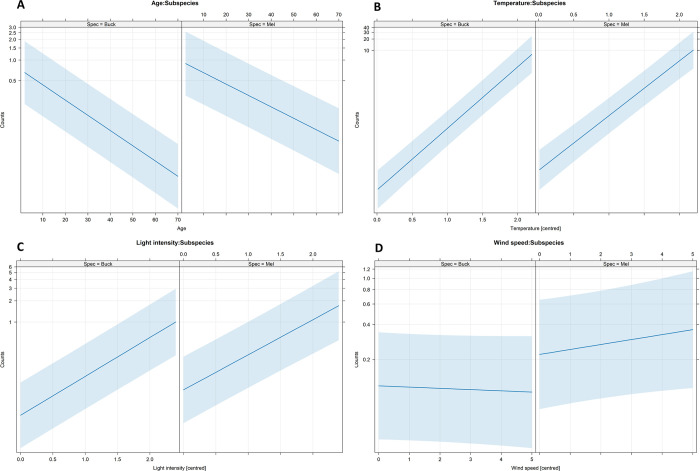
Effect plots of the covariances used to determine differences between *Buck* and *Mel* drones. The effect of A) age, B) temperature, C) light intensity, and D) wind speed in interaction with subspecies on the number of hourly registrations for *Buck* and *Mel* drones. *Mel* showed significantly higher hourly counts with age compared to *Buck* [*Buck*: hybrid *Buckfast*, *Mel*: *Apis mellifera mellifera*].

Age, temperature and light intensity were significant covariates, but only age in interaction with subspecies showed a significant effect on the hourly number of registrations (S6 Table). A significant difference between *Mel* and *Buck* in terms of age was found (S7 Table, Fig 2). The zero-inflation term (‘Hour– 1’) showed a significant negative effect (Estimate = -0.062, p < 0.001).

### Lifespan

**Fig 3:**
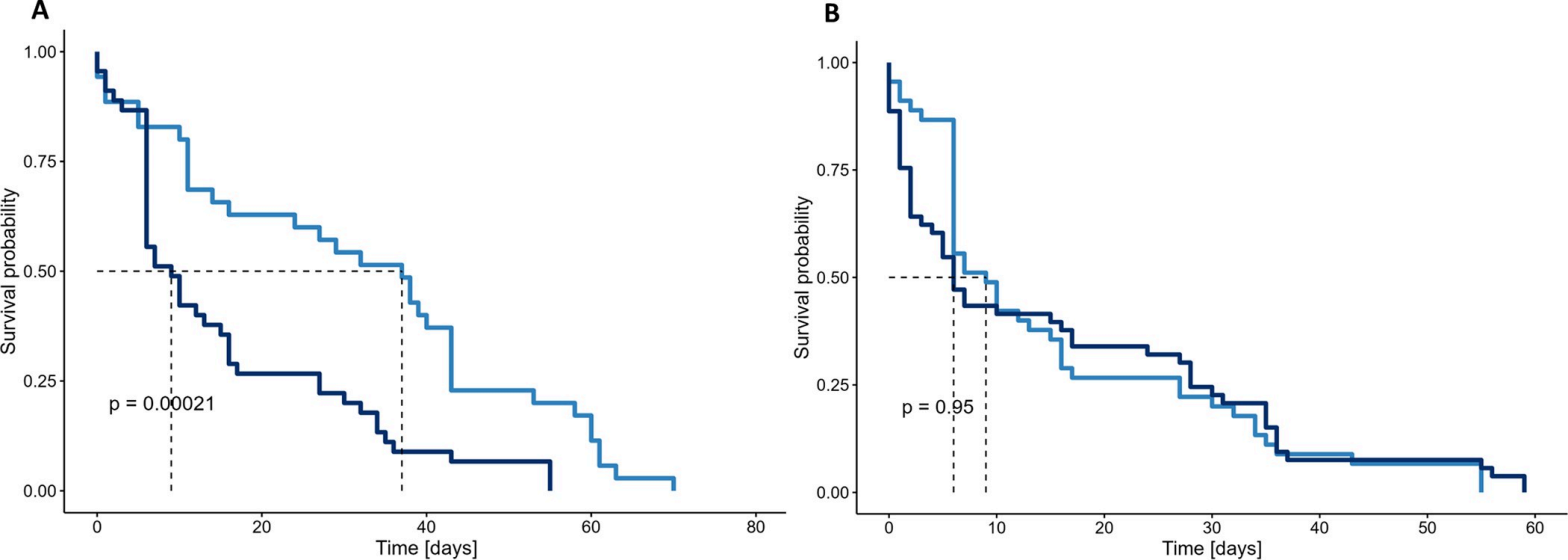
Kaplan-Meier survival curves of drones. A) *Buck* drones’ survival in spring (light blue) and in summer (dark blue), B) *Buck* (light blue) and *Mel* (dark blue) drones’ survival in summer. The survival curves were compared with the log rank test. A significant difference between the survival of spring and summer *Buck* drones was found. No difference was found between *Mel* and *Buck* drones in summer [*Buck*: hybrid *Buckfast*, *Mel*: *Apis mellifera mellifera*].

The mean age of drones at their last registration was 19.8 days (Standard error (SE) = 1.7, n = 133) with a maximal lifespan of 70 days (n = 1). *Buck* drones tagged in spring survived significantly longer than *Buck* drones tagged in summer (p < 0.001, Chisq = 13.7), 31.5 days (Standard deviation (SD) = 21.6, n = 35) compared to 15.8 days (SD = 15.4, n = 45) (Fig 3A). *Mel* drones tagged in summer survived on average 15.6 days (SD = 17.6, n = 53) (Fig 3B). *Buck* drones survived at most 70 and 55 days in spring and summer, respectively, whereas *Mel* drones showed a maximal lifespan of 59 days in summer. The GLMM revealed that both *Buck* and *Mel* drones showed significantly less registrations with increasing age, with a significantly lower negative effect in *Mel* in relation to *Buck* (p < 0.001, S7 Table, Fig 2).

### Age at first activity

**Fig 4:**
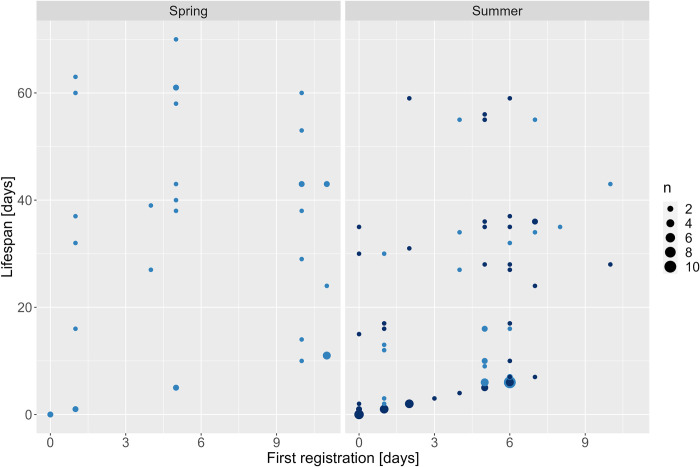
Age at first registration (days) and lifespan (days) of *Buck* (light blue) and *Mel* (dark blue) drones tagged in spring and summer. The size of the points is based on their occurrence in the dataset (i.e., the larger the point the more occurrences) [*Buck*: hybrid *Buckfast*, *Mel*: *Apis mellifera mellifera*].

The median (Mdn) age of drones at first registration was five days (Interquartile Range (IQR) = 5) and the latest first registration happened after 11 days (n = 7) (Fig 4). The Wilcoxon test showed that the median age at first activity differed significantly between spring (Mdn = 5, IQR = 7.5) and summer (Mdn = 5, IQR = 5) individuals (p = 0.022, W = 2159, n_Spring_ = 35, n_Summer_ = 98). The median age at first registration of *Buck* drones (Mdn = 5, IQR = 2) was significantly greater than that of *Mel* drones (Mdn = 3, IQR = 5) in summer (p = 0.014, W = 1497.5, n_Buck_ = 45, n_Mel_ = 53).

### Diurnal activity

**Fig 5:**
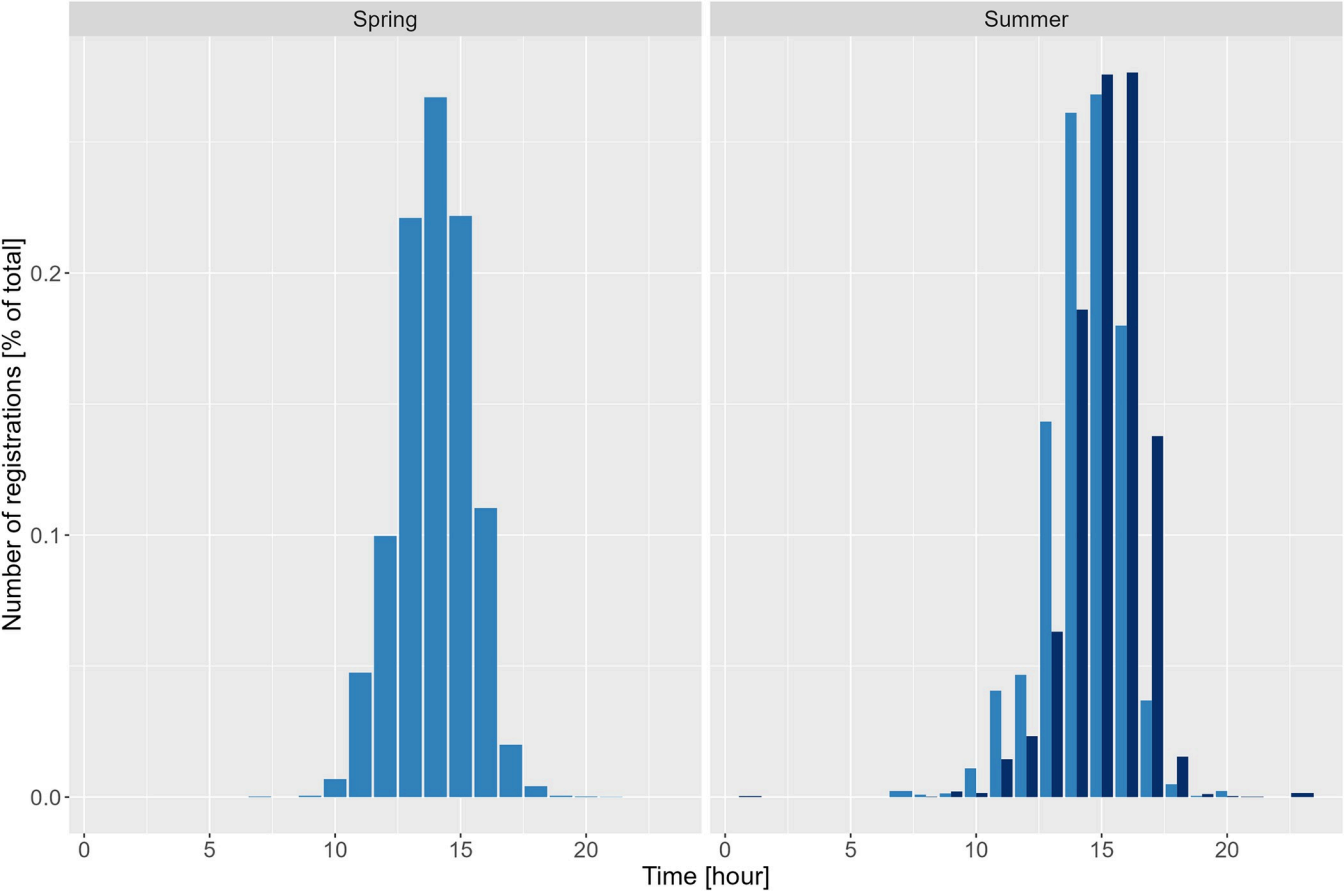
Relative number of *Buck* (light blue) and *Mel* (dark blue) drone registrations in spring and summer. Relative numbers are here presented as the relation between the number of registrations per hour to the total number of registrations. *Mel* showed a later peak activity than *Buck* in summer. [*Buck*: hybrid *Buckfast*, *Mel*: *Apis mellifera mellifera*].

The ANOVA only revealed a significant difference between the average number of registrations by time interval in summer (F(3) = 6.1, p < 0.001) with MORNING and NIGHT showing significant differences to MIDDAY and EVENING. Further, the GLMM revealed that during the first and last time interval significantly fewer registrations occurred. In spring and summer, most registrations of drones occurred during MIDDAY (11 AM—4 PM) (86% and 68%, respectively (S3 Fig)). In summer, more registrations were captured during EVENING (4 PM—9 PM) than in spring (30% *vs*. 13%, respectively), whereby *Mel* drones showed higher relative counts as opposed to *Buck* drones (43% and 22%). *Buck* drones showed a peak in activity between 2 PM and 3 PM in spring and between 2 PM and 4 PM in summer, while in summer *Mel* drones’ peak activity time was between 3 PM and 5 PM (Fig 5).

### Weather influence

**Fig 6:**
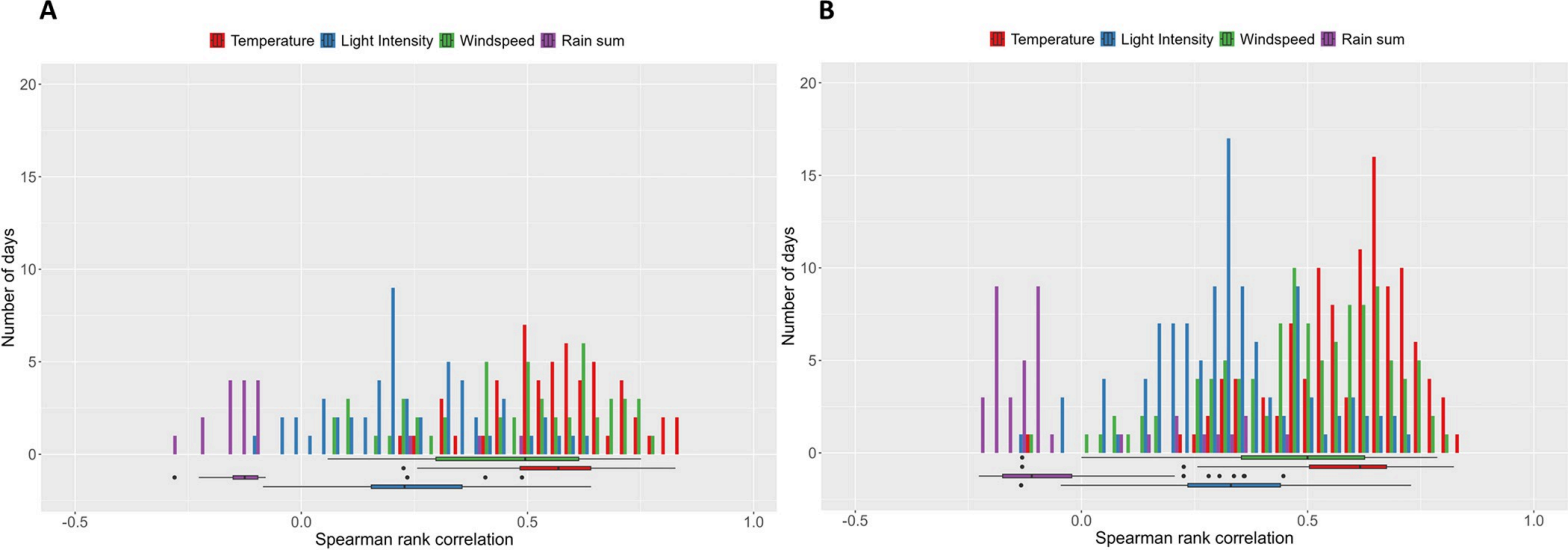
Correlations of the daily number of registrations against environmental parameters. Daily spearman’s rank correlation of the number of registrations of A) *Mel* and B) *Buck* against temperature in red, light intensity in blue, wind speed in green and rain sum in purple. The boxplots at the bottom show the median (line), interquartile range (box) and the top and bottom one percentile (whiskers) for each weather parameter. Outliers are marked with black points [*Buck*: hybrid *Buckfast*, *Mel*: *Apis mellifera mellifera*].

The correlations between the hourly number of registrations per day and temperature and wind speed, respectively, indicate strong correlations [48] (S8 Table, Fig 6). Light intensity and rain sum showed a moderate positive and weak negative average correlation to the hourly number of registrations per day (median), respectively, which means that the drones rarely left the hive when it was raining.

Temperature and light intensity both showed a significant positive effect on the hourly number of registrations in the GLMM (p < 0.001, S6 Table). *Mel* showed more registrations at high light intensities, temperatures and wind speeds in relation to *Buck*, however not significantly (S7 Table, Fig 2).

### Flight length

Drones (n = 72) performed flights of different durations (S4 Fig). Most flights had a length of less than three minutes (35%) and were present over the entire study period. In spring, the median length of flight (Mdn = 11.26, IQR = 26.33) was significantly shorter than in summer (Mdn = 24.55, IQR = 36.62) (p < 0.001, W = 848593). The median length of flight was significantly shorter for *Buck* (Mdn = 14.65, IQR = 28.75) than for *Mel* drones (Mdn = 33.82, IQR = 38.63) in summer (p < 0.001, W = 124935). In both seasons, orientation flights were performed from an early age, starting directly after tagging (S5 and S6 Figs). In spring, *Buck* drones performed orientation flights before starting to perform mating flights. Longer mating flights started to become more frequent at a higher age. In summer, *Mel* and *Buck* drones started to perform mating flights at the same time as orientation flights, already within a few days after tagging. In total, a significant, but weak, positive correlation between the age and flight length was found (Spring: p < 0.001, r = 0.243, Summer: p < 0.001, r = 0.165). The daily peak of shorter flights occurred before the peak of longer flights (S7 Fig), while long mating flights reached their peak when most registrations were made (see diurnal activity).

The analysed comparison of *Mel* and *Buck* drones is summarised in Table 2.

**Table 2:** Differences and similarities between *Buck* and *Mel* drones in summer.

	Difference	*Apis mellifera x (Buck)*	*Apis mellifera mellifera (Mel)*
**Lifespan**	No	55 days (15.8 ± 15.4)	59 days (15.6 ± 17.6)
**Age at first activity**	Yes	Later (Mdn = 5, IQR = 2)	Earlier (Mdn = 3, IQR = 5)
**Diurnal activity**	No	Mostly active during MIDDAY	Mostly active during MIDDAY
Temperature*	No	Positive effect	Positive effect
Light intensity*	No	Positive effect	Positive effect
Wind speed*	No	Negative effect	Positive effect
**Rain**	No	Negative	Negative
**Flight length**	Yes	Shorter (Mdn = 14.65, IQR = 28.75)	Longer (Mdn = 33.82, IQR = 38.63)

Only significant differences between the subspecies are denoted as ‘Yes’ (p < 0.05) [*: parameter tested in the GLMM].

### Comparison with worker bees

RFID registrations of worker bee activity were recorded from the 18^th^ of May 2022 until the 16^th^ of July 2022. Out of 158 tagged worker bees, at least one registration was captured for 110 worker bees (69.6%). 25 worker bees only got registered once. In contrast to drones, all worker bees with registered activity within the first four days of tagging did not show further registrations. In contrast to drones, worker bees’ median number of registrations was only 5.5 (IQR = 74.25).

### Model selection

The collinearity between the weather parameters (S9 Table) did not affect the confidence in the coefficients of the GLMM as all VIFs were less than three [45]. In contrast to the drone GLMM, the worker bee GLMM that also included the interaction between subspecies and rain resulted in the lowest AIC (S10 Table). The residuals of the model showed an acceptable fit (S8 Fig) and the zero-inflation term revealed a negative effect (Estimate = -0.059, p < 0.001). As for the drones, some covariates revealed significant differences (S11 and S12 Tables, S9 Fig for more details).

#### Lifespan

**Fig 7:**
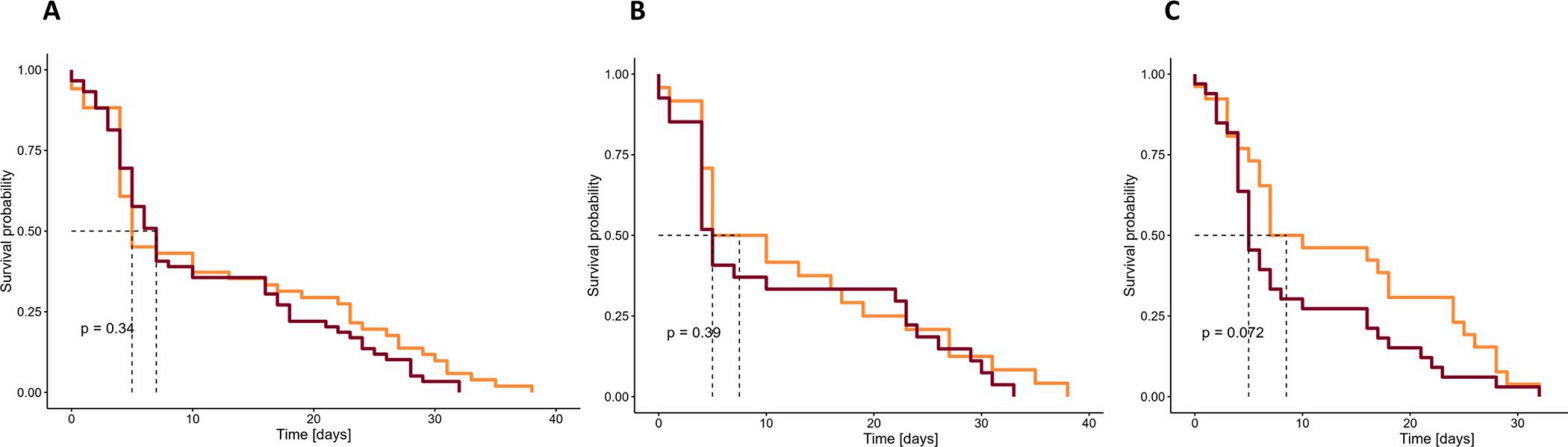
Kaplan-Meier survival curves of worker bees. Worker bees’ survival A) in spring (light orange) and summer (dark orange), of *Buck* (light orange) and *Mel* (dark orange) in B) spring and C) summer. No significant difference between the survival curves was found with the log rank test. *[Buck*: hybrid *Buckfast*, *Mel*: *Apis mellifera mellifera]*.

Worker bees showed a significantly shorter lifespan compared to drones (p < 0.001, Chisq = 22.5) with a mean lifespan of 11.6 days (SE = 0.98, n = 110) and a maximal lifespan of 38 days (n = 1). No difference was found in the worker bee survival between the seasons and subspecies (Fig 7).

The GLMM also revealed that worker bees showed significantly less registrations with increasing age (p < 0.001). In contrast to drones, age showed a significantly higher negative effect on *Mel* worker bees (p = 0.001, CI = [-0.03, -0.01], S12 Table).

#### Age at first activity

**Fig 8:**
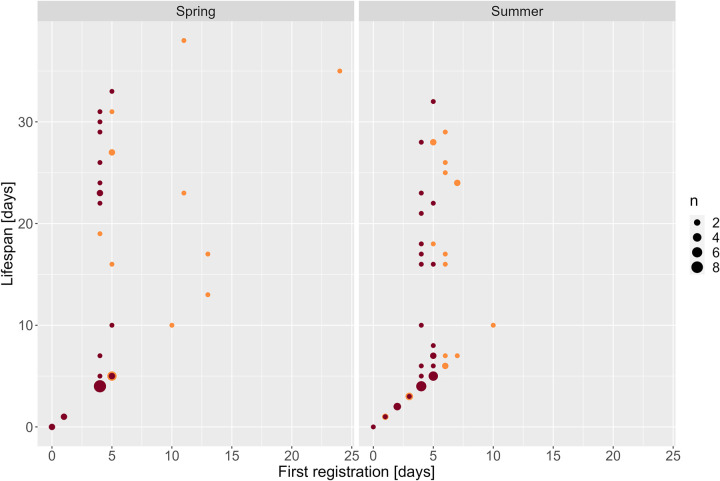
Age at first registration (days) and lifespan (days) of *Buck* (light orange) and *Mel* (dark orange) worker bees tagged in spring and summer. The size of the points is based on their occurrence in the dataset (i.e., the larger the point the more occurrences). All worker bees with registered activity within the first four days of tagging did not show further registrations. *[Buck*: hybrid *Buckfast*, *Mel*: *Apis mellifera mellifera]*.

The median age of worker bees at first registration was four days (IQR = 1) and the latest occurred after 24 days (n = 1) (Fig 8). A significant difference between *Mel* and *Buck* worker bees was observed in both seasons (p < 0.05). Like the drones, *Buck* worker bees showed significantly later first registrations compared to *Mel* worker bees with a median of five (IQR = 1.75) and four (IQR = 1) days, respectively.

#### Diurnal activity

As opposed to drones, worker bees showed registrations throughout the entire day (S3 Fig). However, most registrations of worker bees also occurred during MIDDAY. A significant difference in the average number of registrations by individual type (F(1) = 215.3, p < 0.001), time interval (F(3) = 11, p < 0.001) and their interaction (F(3) = 7.7, p < 0.001) was found. The post hoc analysis revealed that drones showed a significantly higher average number of registrations during MIDDAY and EVENING time intervals (p = 0.001) (S10 Fig). *Mel* and *Buck* worker bees showed similar relative numbers of registrations during the day.

#### Weather influence

The correlations between temperature, light intensity and wind speed and the number of registrations for each day revealed average (median) positive correlations [48] (S13 Table, S11 Fig). Temperature and light intensity both showed a significant positive effect on the hourly number of registrations in the GLMM (p < 0.001, S11 Table). Only temperature revealed a significant difference between *Buck* and *Mel* worker bees with a lower positive effect on *Mel* worker bees in relation to *Buck* (p = 0.011, S12 Table).

#### Flight length

Worker bees (n = 47) showed numerous short flights, both in spring and summer, as well as extremely long flights during summertime (S12 Fig). The median length of flight of drones (Mdn = 18.55, IQR = 30.33) was significantly shorter than of worker bees (Mdn = 14.53, IQR = 67.79) (p < 0.001, W = 1230980). In summer, worker bees mostly performed very long flights (> 60 min) (36.2%), followed by shorter flights (3–10 min) (16%) while intermediate flight lengths rarely occurred (5.7% and 7.7%, respectively). In both seasons the flight length increased significantly with age (Spring: p < 0.001, r = 0.298, Summer: p < 0.001, r = 0.344).

The analysed comparison of drones and worker bees is summarised in Table 3.

**Table 3:** Differences and similarities between drones and worker bees.

	Difference	Drones	Worker bees
**Lifespan**	Yes	70 days (19.8 ± 19.2)	38 days (11.6 ± 10.4)
**Age at first activity**	No	Mdn = 5 days (IQR = 5)	Mdn = 4 days (IQR = 1)
**Diurnal activity**	Yes	Higher MIDDAY and EVENING activity.	Most activity during MIDDAY, but lower.
**Flight length**	Yes	Shorter (Mdn = 18.55, IQR = 30.33)	Longer (Mdn = 14.53, IQR = 67.79)

Only significant differences are denoted as ‘Yes’ (p < 0.05).

## Discussion

RFID data of honeybee drones was collected for the first time in Sweden. The outcomes have provided insights into various aspects of the ecology of drones with some interesting observations about differences between *Mel* and *Buck* colonies and between drones and worker bees, all tagged at two different times during a Swedish mating season.

To our knowledge, in northern Europe this is the first study focusing on the one hand on drones’ ecology of different *Apis mellifera* subspecies and/or breeds, respectively, and on the other hand monitoring drones over the whole mating season. Overall, our results are mainly comparable to older literature which shows that RFID is a suitable monitoring method for honeybee drones, e.g. [15–19, 23, 24, 26–29].

### Model selection

Four models were developed in order to investigate the effects of the environmental factors on the hourly count data (S5 Table). Only linear combinations of parameters were used in the GLMM models due to the complexity of the analysis. To investigate a wide range of parameters within a complex model framework is already a significant task. Introducing non-linearities could substantially increase the complexity, potentially affecting the interpretability and computational feasibility. However, the chosen approach here enables a detailed examination of linear relationships, providing valuable insights into the interactions between environmental factors, age, and behaviour.

### Lifespan

The lifespan was estimated as the number of days until the last registration, either at their natal or a foreign hive. [49] found no effect of drifting on the survival of bees, hence including such registrations in our study is legitimate to increase the amount of data. Fewer registrations occurred the higher the age. This effect is likely linked to a higher mortality. Drones tagged in spring survived longer than drones tagged in summer, as observed by [50] who hypothesised that this was due to higher flight activity and higher temperatures in summer, as was the case in this study (S3 Table, S7 Fig). The results (spring: 70 ± 31.5 days, summer: 59 ± 15.6 days) differ in length from those reported by [16] (spring: approximately 54 days, n = 12, USA), [52] (summer: mean of 21.2 days, USA), [24] (summer: 45 ± 13.9 days, autumn: 75 ± 32, 90 ± 42.5, 80 ± 39 days, Japan) and [23] (spring: 33 ± 17.9 (n = 90), summer: 21 ± 15.2 days (n = 86), France). The difference between the recorded lifespans of drones in Sweden to other reported lifespans in literature might be due to different methods, geographical regions, subspecies, and seasons used during the studies that led to different experimental and environmental conditions, preventing a direct comparison. No difference in lifespan was found between the *Mel* and *Buck* drones tagged in summer.

### Age at first activity

No relationship between the age at first activity and lifespan could be observed. First registrations of drones were already present at the day of tagging from which 41% did show further registrations during the experiment. This shows that not all early registrations displayed evictions from the hive or, e.g., death, misorientation, during their first activity outside of the hive. Evictions from the hive might have occurred due to the smell of the glue used to fixate the RFID tags on the thorax or drones were sorted out by other hive members due to a difference in appearance caused by the RFID tag [51]. Most drones were five and six days old (plus 0–72 hours age from the tagging time) at the time of their first registration. This coincides with reported first flight activities between four and eight days of age [15, 16, 52]. Earlier first activities were observed in summer which contradicts [23] who reported earlier first flights in the spring trial, possibly due to an already warmer spring in southern France compared to southern Sweden. No *Mel* drones could be tagged in spring which indicates a faster rearing of drones by *Buck* colonies after winter. This could be a mating advantage for *Buck* drones as they are possibly reaching virgin queens earlier than *Mel* drones. This can, among other factors, potentially lead to the disappearance of *Mel* colonies, as observed for the African Honey Bee subspecies *Apis mellifera scutellata* that has largely replaced European Bees in the Americas [53]. *Mel* drones, in contrast, had significantly earlier first registrations in summer than *Buck*. Both observations of *Mel* might be explained by local adaptations to longer winters and shorter summers [31].

### Diurnal activity

Most activity occurred during MIDDAY (11 AM– 4 PM), but activity could also be observed during the MORNING, EVENING and NIGHT, as reported by [19]. In summer, *Mel* drones showed most activity between 2 PM and 6 PM while most *Buck* activity was observed one hour earlier (between 1 PM and 5 PM) (Fig 5). Tagged drones in Argentina had their highest activity between 2 PM and 5 PM [19]. In southern France, most activity was observed between 2 PM and 6 PM in spring and 2 PM and 7 PM in summer with a peak at 4 PM during both seasons, indicating seasonal differences with a longer time range of activity in summer [23]. In Sweden, no such differences were found for the *Buck* drones. The observed shift in activity between *Buck* and *Mel* might be caused by a local adaptation of *Mel* to the longer days with high light intensities, especially on and around midsummer (21^st^ of June). Further studies comparing the flight activity of *Apis mellifera* queens with different genetic backgrounds would be of high interest because possible reproductive isolation could be another explanation [54–56]. From two giant honey bee species reproductive isolation is known [57].

### Weather influence

The study demonstrated that temperature, light intensity, wind speed and rain affected drones’ activity. Temperature and light intensity had a positive effect on *Buck* and *Mel* drones. [23] obtained similar observations and found that drones did not fly when wind speeds exceeded 30 km/h or when it rained. [29] also reported no observations under rainy conditions and positive correlations with temperature and light intensity. A correlation analysis by [19] between the average daily weather data and the mean daily number of drone flights revealed a positive and negative effect of temperature and precipitation, respectively. However, rarely a wind speed of more than 30 km/h was measured during the experiment and rain was only recorded on 36 days with a maximum precipitation of 6.8 mm within one hour (25^th^ of July 2022, S1 Fig, S3 Table), but it can be stated that *Mel* drones flew more in higher wind speeds than *Buck* (Fig 2). [31, 32] stated that *Mel* is well-adapted to the harsher climatic conditions of Northern Europe.

### Flight length

Most flights were less than three minutes long, which were not interpreted as proper flights but rather as quick exits and entries to defecate or orientate. [58] showed that these very short flights occur throughout the entire lifespan of a drone, as also observed during this study (S5 and S6 Figs). According to previous literature, drones stay inside the hive until they begin performing their first cleaning and/or orientation flights at an age between 4–8 days, e.g., [16, 21, 52]. However, flight durations of more than three minutes, even longer than ten minutes, were observed from the day of tagging onwards (S5 and S6 Figs). This supports the assumption that drones perform shorter flights before they start mating flights but contradicts the statement that longer flights are first carried out after sexual maturity (age of 12 days), e.g., [21, 23, 52].

Relatively few flights had a length of 3–10 minutes (orientation flights). In contrast, [19] reported the highest frequency of flights at that length, but monitored drones for 1.5 months only, hence not during the entire mating period. In this study, short mating flights (10–30 min) dominated in spring while longer mating flights (30–60 min) were mostly present in summer (S4 Fig). Higher temperatures and lower wind speeds during summer, could have encouraged longer mating flights. [52] observed that unfavourable weather conditions led to shorter flights. In addition, for successful mating flights, virgin queens must be present and perform nuptial flights. [59] stated that queens prefer temperatures of at least 20°C with little cloud cover. During this study, maximal temperatures rarely reached 20°C after drones have reached sexual maturity in spring (S1A Fig). Further, drones started to perform mating flights in the afternoon, as observed by [60]. In this study, *Buck* drones showed peaks of longer flights earlier during the day than *Mel* drones (S7 Fig). A possible reason for these differences between the subspecies may be that drone mating flights are triggered by different climatic conditions or that the queen of each subspecies prefers different conditions for mating flights, which can cause reproductive isolation.

### Comparison with worker bees

Worker bees lived significantly shorter than drones (max. 38 days *vs*. 70 days for drones). As for the drones, the lifespan was longer in spring, however not significantly. [61] also reported longer lifespans of worker bees in spring (30–40 days) as opposed to summer (25–30 days). The lifespan of worker bees performing different tasks differs, with a lifespan of only 4–5 days for foraging worker bees [3, 59]. This might explain the faster drop in the survival of worker bees as opposed to drones. Any worker bees that showed first registrations up to three days after tagging could have been evicted from the hive, because they were not registered again. Worker bees perform several different tasks throughout their life, where ventilation represents the first task outside of the hive at an age of 18 days, but workers of any age have been found to ventilate [62, 63]. This coincides with the wider range of first registrations of worker bees as opposed to drones (max = 24 *vs*. max = 11 days). As for drones, no relationship between the age at first registration and lifespan could be observed, representing individual lifespans of the tagged bees. [64] indicated that worker bees with lower daily foraging activity reach energy deficiency and subsequently die later than worker bees with higher foraging requirements.

Most registrations of worker bees were captured during MIDDAY, as was the case for drones. However, drones were on average significantly more active during MIDDAY and EVENING, while worker bees were active during the entire day (S10 Fig, Table 3). Also, [23] described that worker bees’ peak activity changed daily. The weather conditions might have triggered this behaviour in worker bees, but in contrast to drones, worker bees showed a higher activity on rainy days (however more than 10 mm of rain per day was rarely recorded during the entire study, S1C Fig). The higher activity of worker bees could be explained by their duty to forage while drones might be aware that no mating flights occur under such conditions.

The flight length of worker bees is hard to interpret because it is not known which tasks the tagged bees were responsible for. However, as opposed to drones, they performed more flights lasting longer than one hour suggesting most worker bees were foraging nectar. [59] observed that nectar collectors take longer trips (30–80 min) as opposed to pollen collectors (around 10 min). The high proportion of short flights at an early age could represent worker bees performing orientation flights before beginning to forage, because it is known that such flights only take up to five minutes [59].

### Technical considerations

RFID registrations during rainy weather confirmed that the system operated well under wet conditions. However, the RFID technology had its limitations in terms of misidentification of the movement direction, possibly missing registrations, and the dependency on continuous technical check-ups. Additionally, a high proportion of tagged drones and worker bees got lost due to, e.g., ejection through other colony members, loss of the tag or other unknown reasons. Such limitations were also stated by [19]. Further, successful mating flights could not be registered completely (without arrival) because drones die directly after mating with a virgin queen [65]. Frequent unknown and possibly misidentified registrations restricted the analysis of the flight activity because they could not be subsequently replaced by arrivals or departures. Not only the misidentification of the movement direction itself produced false registrations but also drones crawling underneath or coming from underneath the entrance were registered. In fact, flights could be identified for only half of the tagged bees with at least one registration. The departure-arrival sequences defined as a flight can thus contain errors and the results must be interpreted cautiously. This is also the reason why the activity at the entrance of the hives was mainly analysed instead of the flight activity. Hereby we included drifting registrations to enlarge the dataset and to allow for a more precise analysis. A future study with an optimized RFID setup would provide further insight into drone ecology of different honeybee subspecies around the world.

## Conclusion

To our knowledge no study on the ecology of drones has yet been conducted in Sweden nor has any previous study focused on the differences between *Apis mellifera* subspecies in northern Europe. In contrast to most recent studies on honeybee drones [19, 23], *Buck* and *Mel* bees were monitored throughout their entire life using RFID technology. This continuous tracking of tagged bees at the entrance of the hives provided novel information about drone and worker bee ecology in Sweden. As previously reported, drones survived longer in spring than in summer as well as longer than worker bees, drones show mainly first activities around the entrance of the hive between four and eight days of age, but also earlier and later first registrations occurred. To fully capture the age at first activity, only freshly hatched individuals would have to be tagged, but the results here indicated a tendency of first activity outside the hive already at an earlier age than described in literature. Additionally, this study observed that as soon as orientation flights were started, mating flights also occurred. This contradicts the widely accepted knowledge that drones only start to perform mating flights as soon as they reach sexual maturity. Drones were mainly active between 11 AM and 5 PM with peak activity times in the afternoon. Registrations during earlier and later hours occurred, as previously shown by [19, 23], both also using automated monitoring methods. Further studies are needed to observe drones’ behaviour at those times together with the monitoring of virgin queens to know when mating flights occur in northern Europe. Furthermore, drones preferred to fly when temperatures and light intensities were high and when there was no rain, which is particularly different from worker bees. Differences between *Buck* and *Mel* were found, e.g., in terms of the time of drone production (*Mel* later in spring), age at first activity (*Mel* earlier for drones and worker bees), diurnal activity (*Mel* drones show higher activity later in the day), weather preferences (*Mel* more active during windy conditions) and flight length (*Mel* drones and worker bees flew longer) (Tables 2 and 3). These observed differences give support for local adaptations of *Mel* colonies, as reported by [31, 32, 34, 35]. Until now, such observations have been rare, however they call for an urgent need to increase conservational efforts of the native but threatened *Apis mellifera mellifera* to preserve important regional and local traits in Sweden [66] and other northern European countries.

## Supporting information

S1 Fig Time series of the weather parameters analysed. Daily A) average temperature (°C), B) average wind speed (km/h), C) rain (mm), D) humidity (%) and E) average light intensity.
(TIF)

S2 Fig DHARMa residual plots for the generalised linear mixed model of drones. A) QQ residual plot and B) residual *vs*. predicted plot. The Kolmogorow-Smirnow-Test is significant in drones’ data, but it does not appear to have a large effect and is likely an effect of the large data set.
(TIF)

S3 Fig Number of registrations of all drones (blue) and all worker bees (red) in spring and summer by time interval, independent of subspecies. Relative numbers are here presented as the relation between the number of registrations per time category to the total number of registrations. Drones (blue) and worker bees (red) showed most registrations during MIDDAY. Worker bees show relatively more registrations during MORNING, EVENING and NIGHT compared to drones. In summer, the proportion of EVENING registrations was higher than in spring. For worker bees relatively more MORNING registrations occurred in spring.
(TIF)

S4 Fig Number of flights of *Buck* (light blue) and *Mel* (dark blue) drones in spring and summer per length category. Relative numbers are given, thus the relation between the number of flights in a length category to the total number of flights. In summer, *Mel* performed relatively more longer flights *[Buck*: hybrid *Buckfast*, *Mel*: *Apis mellifera mellifera]*.
(TIF)

S5 Fig Number of flights of *Buck* drones in spring. A) Total and B) relative number of flights over time grouped by the flight length category for *Buck* drones in spring *[Buck*: hybrid *Buckfast]*.
(TIF)

S6 Fig Number of flights of *Buck* and *Mel* drones in summer. A) Total and B) relative number of flights over time grouped by the flight length category for *Buck* and *Mel* drones in summer *[Buck*: hybrid *Buckfast*, *Mel*: *Apis mellifera mellifera]*.
(TIF)

S7 Fig Length of flights by hour for *Buck* and *Mel* drones in spring and summer. Relative numbers are given, thus the relation between the number of flights per hour to the total number of flights. The darker the colour palette, the longer the flight length. Longest flight lengths (mating flights) occurred during high activity times in the afternoon [*Buck*: hybrid *Buckfast*, *Mel*: *Apis mellifera mellifera*].
(TIF)

S8 Fig DHARMa residual plots for the generalised linear mixed model of worker bees. A) QQ residual plot and B) residual *vs*. predicted plot. A) and B) indicate an acceptable fit of the model.
(TIF)

S9 Fig Effect plots of the covariances used to determine differences between *Buck* and *Mel* worker bees. The effect of A) age, B) temperature, C) light intensity, D) wind speed and E) rain in interaction with subspecies on the number of hourly registrations for *Buck* and *Mel* worker bees. Significant differences were found in A) and B) [*Buck*: hybrid *Buckfast*, *Mel*: *Apis mellifera mellifera*].
(TIF)

S10 Fig Number of registrations grouped by individual type and time interval. This figure shows the visualisation of the post hoc test of the ANOVA. The filled points display the mean, and the error bars show the standard error. The red letters indicate significant differences. Drones showed significantly more registrations during MIDDAY and EVENING than worker bees.
(TIF)

S11 Fig Daily correlation of the number of registrations of worker bees against environmental parameters. Daily spearman’s rank correlation of the number of registrations of A) *Buck* and B) *Mel* against temperature in red, light intensity in blue, wind speed in green and rain sum in purple. The boxplots at the bottom show the median (line), interquartile range (box) and the top and bottom one percentile (whiskers) for each weather parameter. Outliers are marked with black points [*Buck*: hybrid Buckfast, *Mel*: *Apis mellifera mellifera*].
(TIF)

S12 Fig Number of flights of worker bees in spring and summer. Relative numbers are given, thus the relation between the number of flights in a length category to the total number of flights. In summer, relatively more longer flights were performed by worker bees.
(TIF)

S1 Table Information about the breeders of the queens used in the experiment. *Buck*: hybrid *Buckfast*; *Mel*: *Apis mellifera mellifera*.
(DOCX)

S2 Table Number of tagged drones and worker bees in spring and summer. *Buck*: hybrid *Buckfast*; *Mel*: *Apis mellifera mellifera*.
(DOCX)

S3 Table Monthly overview (May–August, 2022) of the weather parameters analysed. Temp, Temperature (°C); Light, light intensity (PAR); Wind, wind speed (km/h); Rain sum (mm). Humidity was not analysed due to mainly high constant values (S1D Fig).
(DOCX)

S4 Table Collinearity between the weather parameters used in the generalised linear mixed model selection for drones. (DOCX)

S5 Table The different drone generalised linear mixed models’ Akaike information criterium (AIC) values. All model structures contained the same random effects and zero inflation formula. [A, Age; T, Temperature; L, Light intensity; W, Wind speed; R, Rain; S, Subspecies; D, Time interval].
(DOCX)

S6 Table Analysis of deviance of the final generalised linear mixed model of *Buck* and *Mel* drones. Significance codes: p < 0.001 = ***, p < 0.01 = **, p < 0.05 = *, p < 0.1 =. [*Buck*: hybrid *Buckfast*, *Mel*: *Apis mellifera mellifera*].
(DOCX)

S7 Table Parameters of the final generalised linear mixed model of *Buck* and *Mel* drones. The 95% Confidence Intervals (CI) were used to detect significant differences between both subspecies. A significant difference in age was found [*Buck*: hybrid *Buckfast*, *Mel*: *Apis mellifera mellifera*].
(DOCX)

S8 Table Median correlations between the weather parameters and the hourly number of registrations per day for *Buck* and *Mel* drones. *Buck*: hybrid *Buckfast*; *Mel*: *Apis mellifera mellifera* [Mdn: Median, IQR = Interquartile range].
(DOCX)

S9 Table Collinearity between the weather parameters used in the generalised linear mixed model selection for worker bees. (DOCX)

S10 Table The different worker bee generalised linear mixed models’ Akaike information criterium (AIC) values. All model structures contained the same random effects and zero inflation formula. [A, Age; T, Temperature; L, Light intensity; W, Wind speed; R, Rain; S, Subspecies; D, Time interval].
(DOCX)

S11 Table Analysis of deviance table of the final generalised linear mixed model for *Buck* and *Mel* worker bees. Significance codes: p < 0.001 = ***, p < 0.01 = **, p < 0.05 = *, p < 0.1 = .
(DOCX)

S12 Table Parameters of the final generalised linear mixed model for worker bees. The 95% Confidence Intervals (CI) were used to detect significant differences between both subspecies. Significant differences in age and temperature were found [*Mel*: *Apis mellifera mellifera*].
(DOCX)

S13 Table Median correlations between the weather parameters and the number of registrations for each day. *Buck*: hybrid *Buckfast*, *Mel*: *Apis mellifera mellifera* [Mdn: Median, IQR = Interquartile range].
(DOCX)
