# Supplementary figures and images for: Ecological comparison of native (Apis mellifera mellifera) and hybrid (Buckfast) honeybee drones in southwestern Sweden indicates local adaptation
Source: PLoS One. 2024 Aug 13;19(8):e0308831. doi: 10.1371/journal.pone.0308831 (PMC11321565; doi:10.1371/journal.pone.0308831)

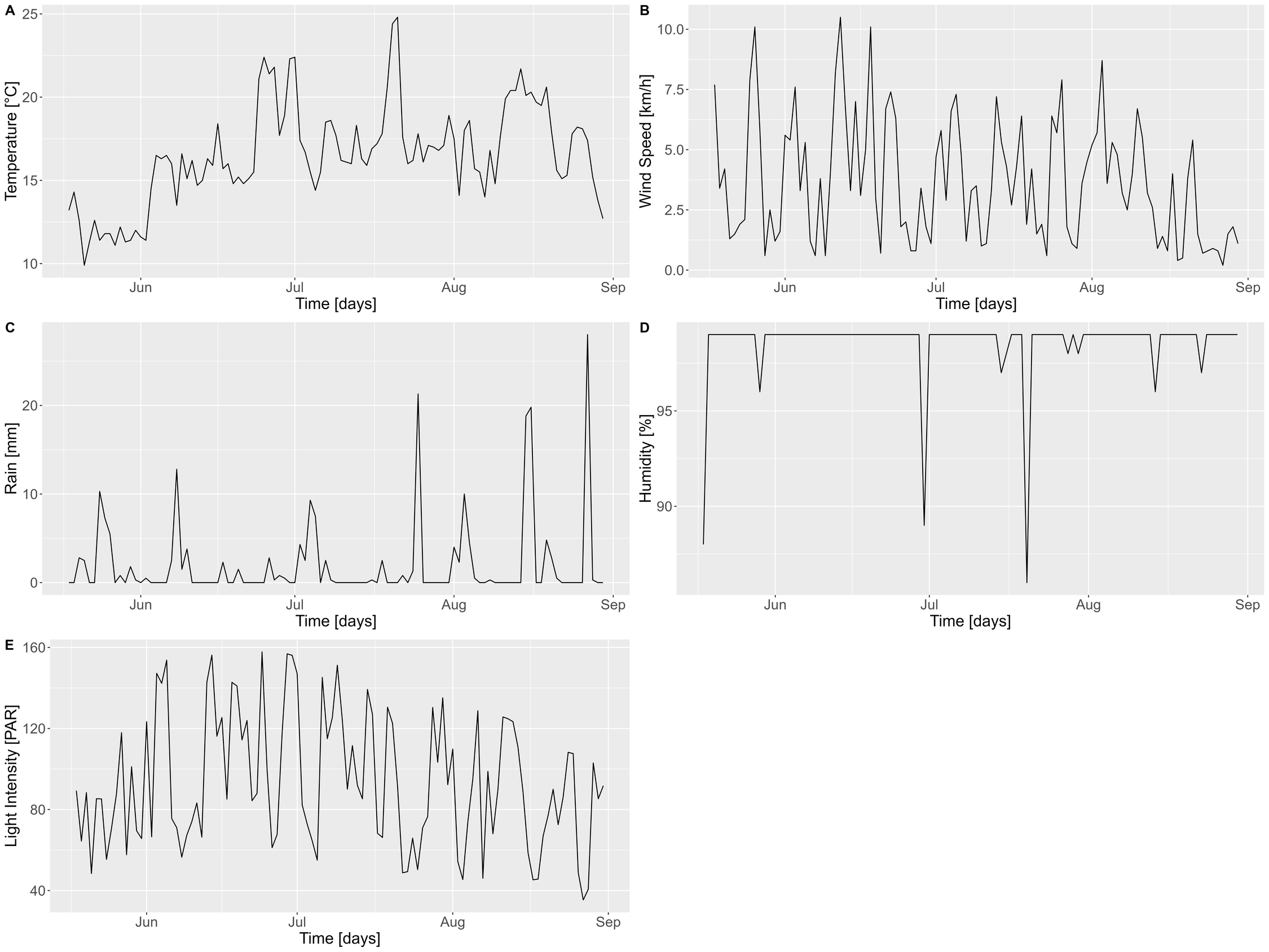

Supplement: S1 Fig — Daily A) average temperature (°C), B) average wind speed (km/h), C) rain (mm), D) humidity (%) and E) average light intensity. (TIF) [file pone.0308831.s001.tif]

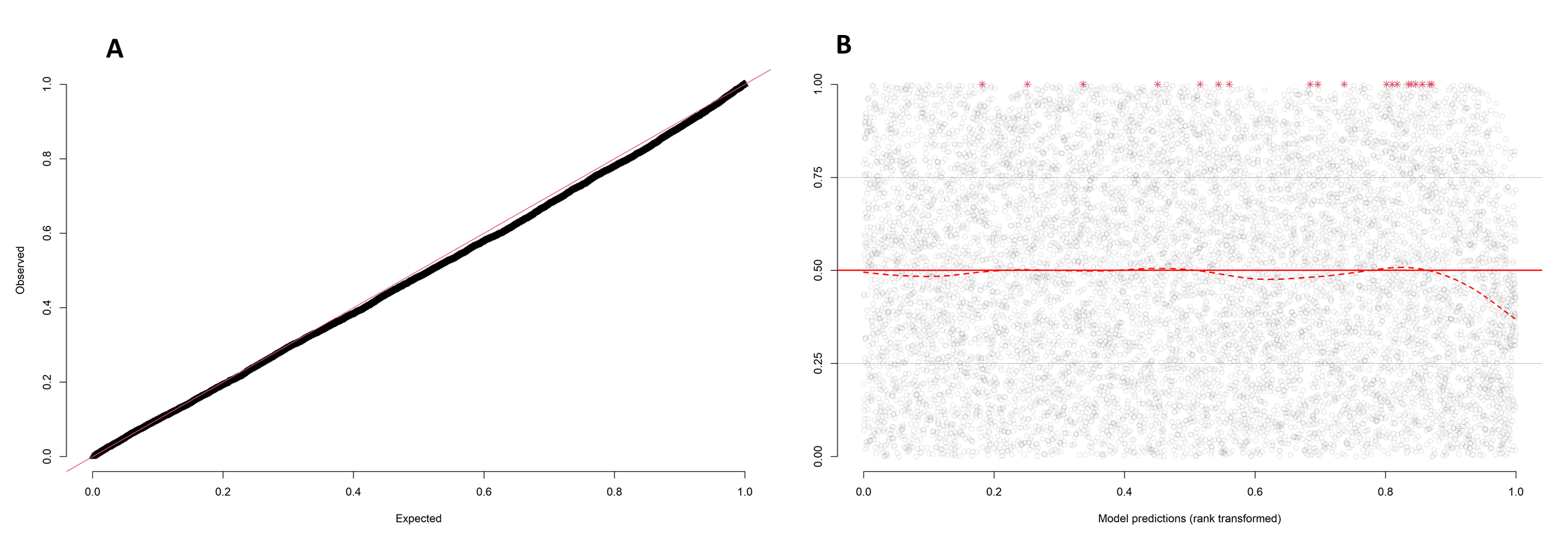

Supplement: S2 Fig — A) QQ residual plot and B) residual vs. predicted plot. The Kolmogorow-Smirnow-Test is significant in drones’ data, but it does not appear to have a large effect and is likely an effect of the large data set. (TIF) [file pone.0308831.s002.tif]

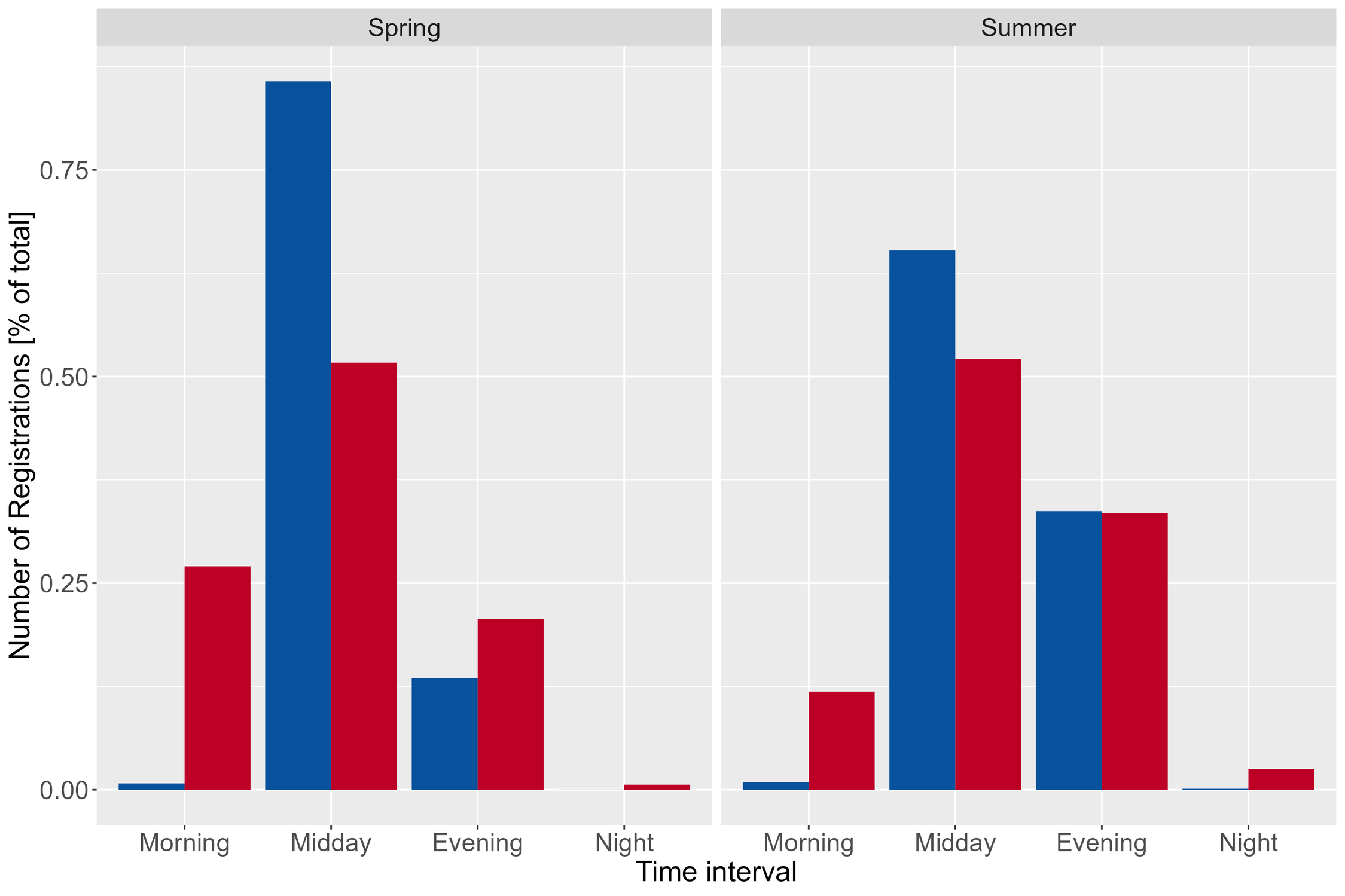

Supplement: S3 Fig — Relative numbers are here presented as the relation between the number of registrations per time category to the total number of registrations. Drones (blue) and worker bees (red) showed most registrations during MIDDAY. Worker bees show relatively more registrations during MORNING, EVENING and NIGHT compared to drones. In summer, the proportion of EVENING registrations was higher than in spring. For worker bees relatively more MORNING registrations occurred in spring. (TIF) [file pone.0308831.s003.tif]

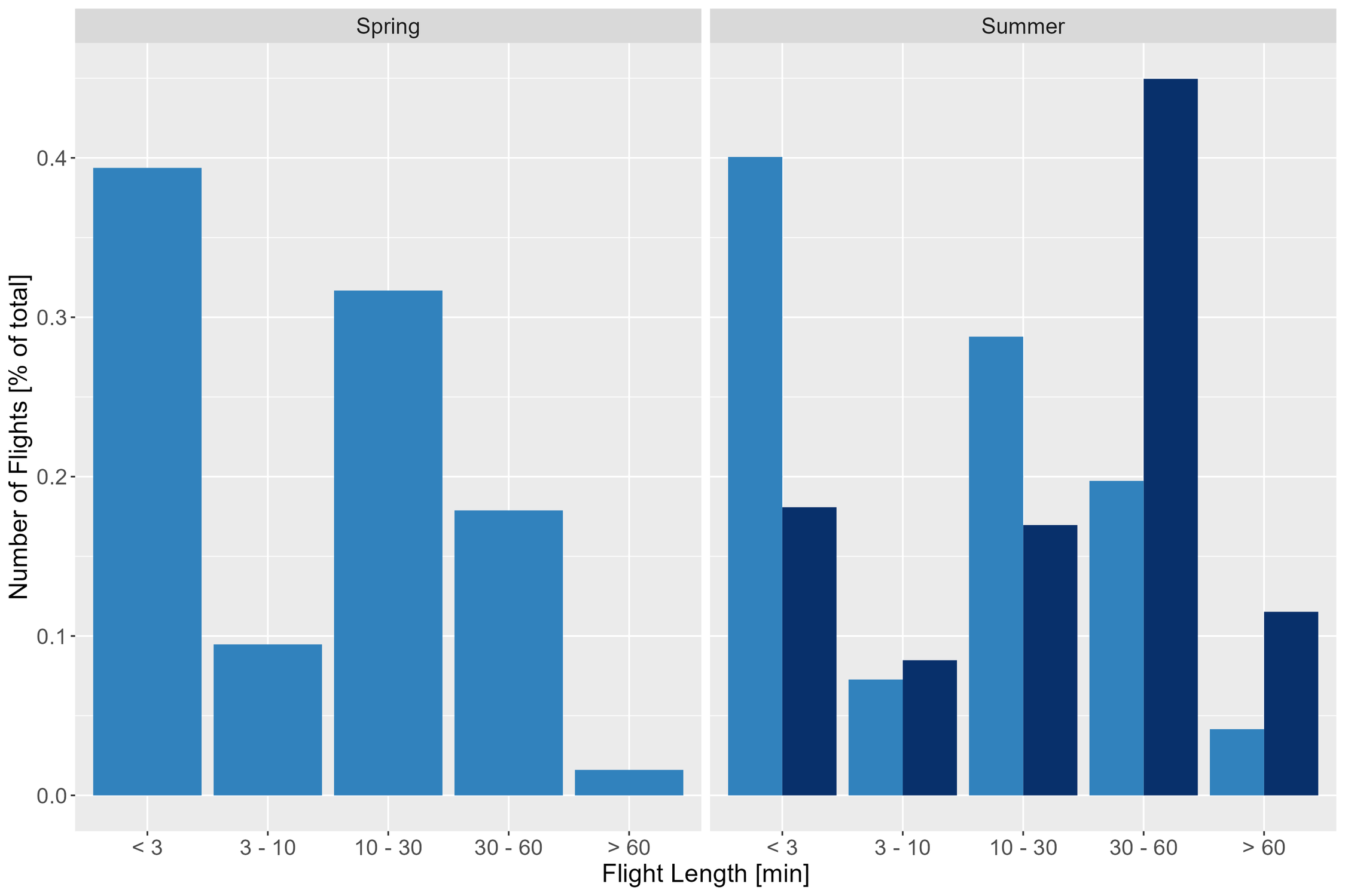

Supplement: S4 Fig — Relative numbers are given, thus the relation between the number of flights in a length category to the total number of flights. In summer, Mel performed relatively more longer flights [Buck: hybrid Buckfast, Mel: Apis mellifera mellifera]. (TIF) [file pone.0308831.s004.tif]

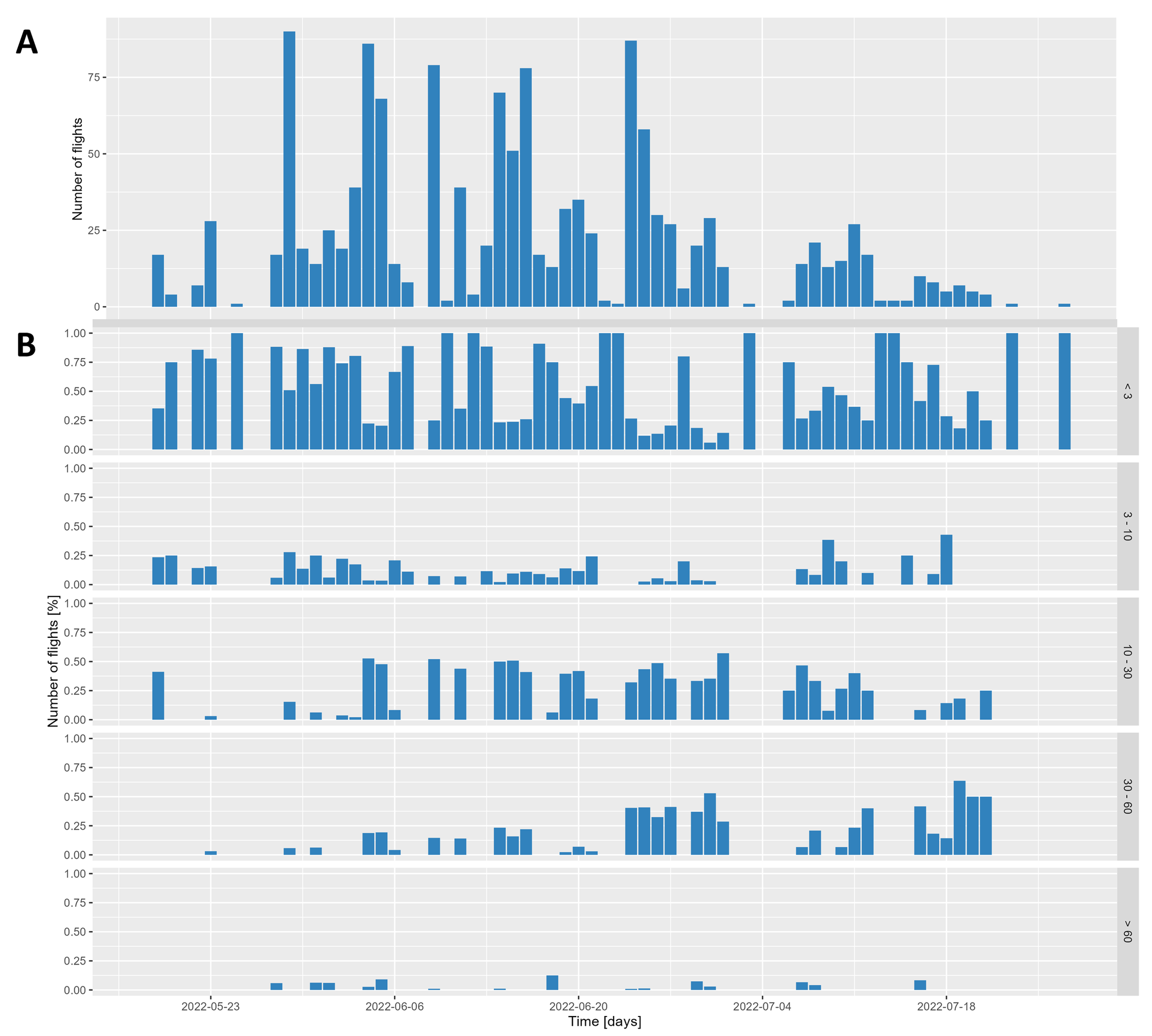

Supplement: S5 Fig — A) Total and B) relative number of flights over time grouped by the flight length category for Buck drones in spring [Buck: hybrid Buckfast]. (TIF) [file pone.0308831.s005.tif]

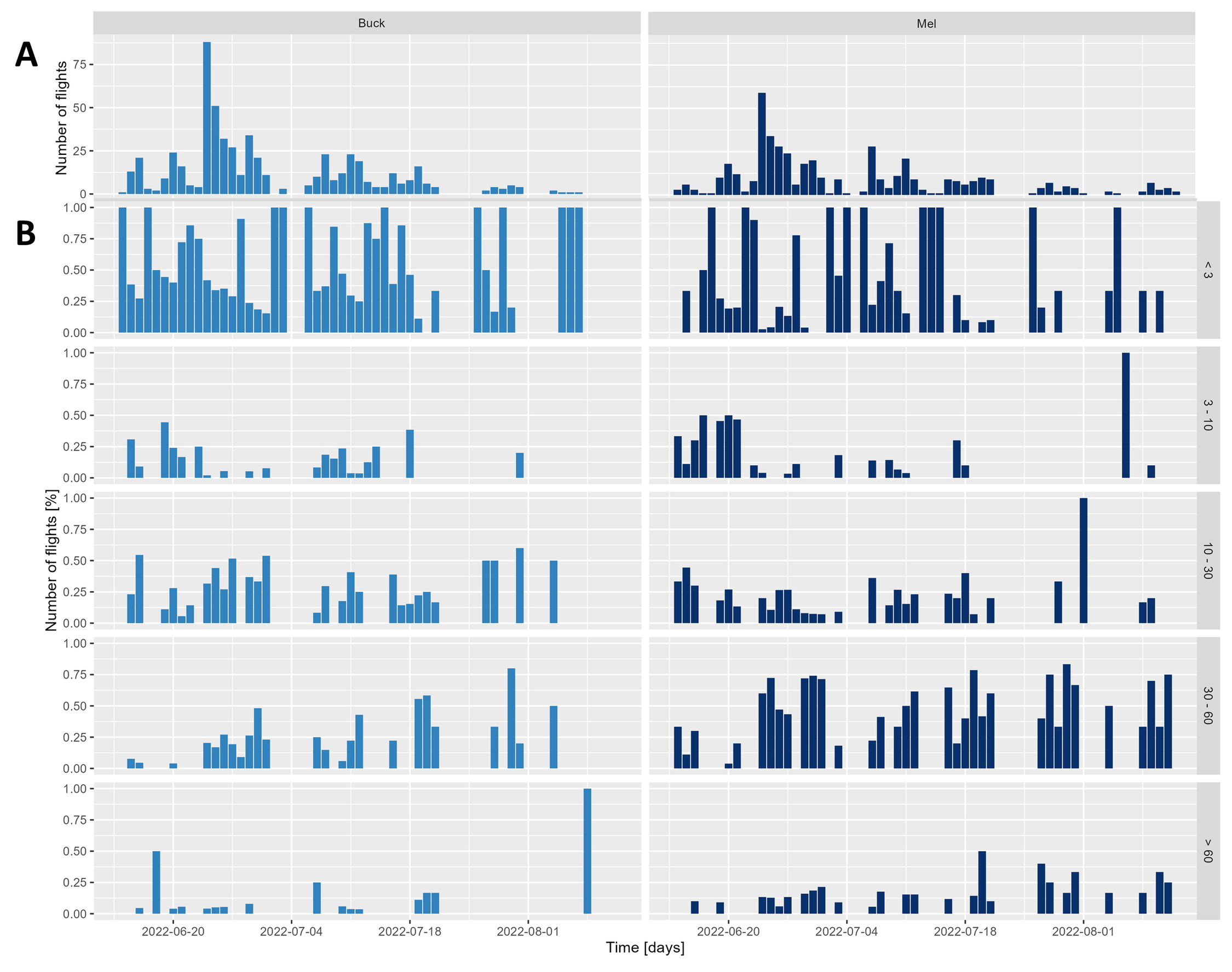

Supplement: S6 Fig — A) Total and B) relative number of flights over time grouped by the flight length category for Buck and Mel drones in summer [Buck: hybrid Buckfast, Mel: Apis mellifera mellifera]. (TIF) [file pone.0308831.s006.tif]

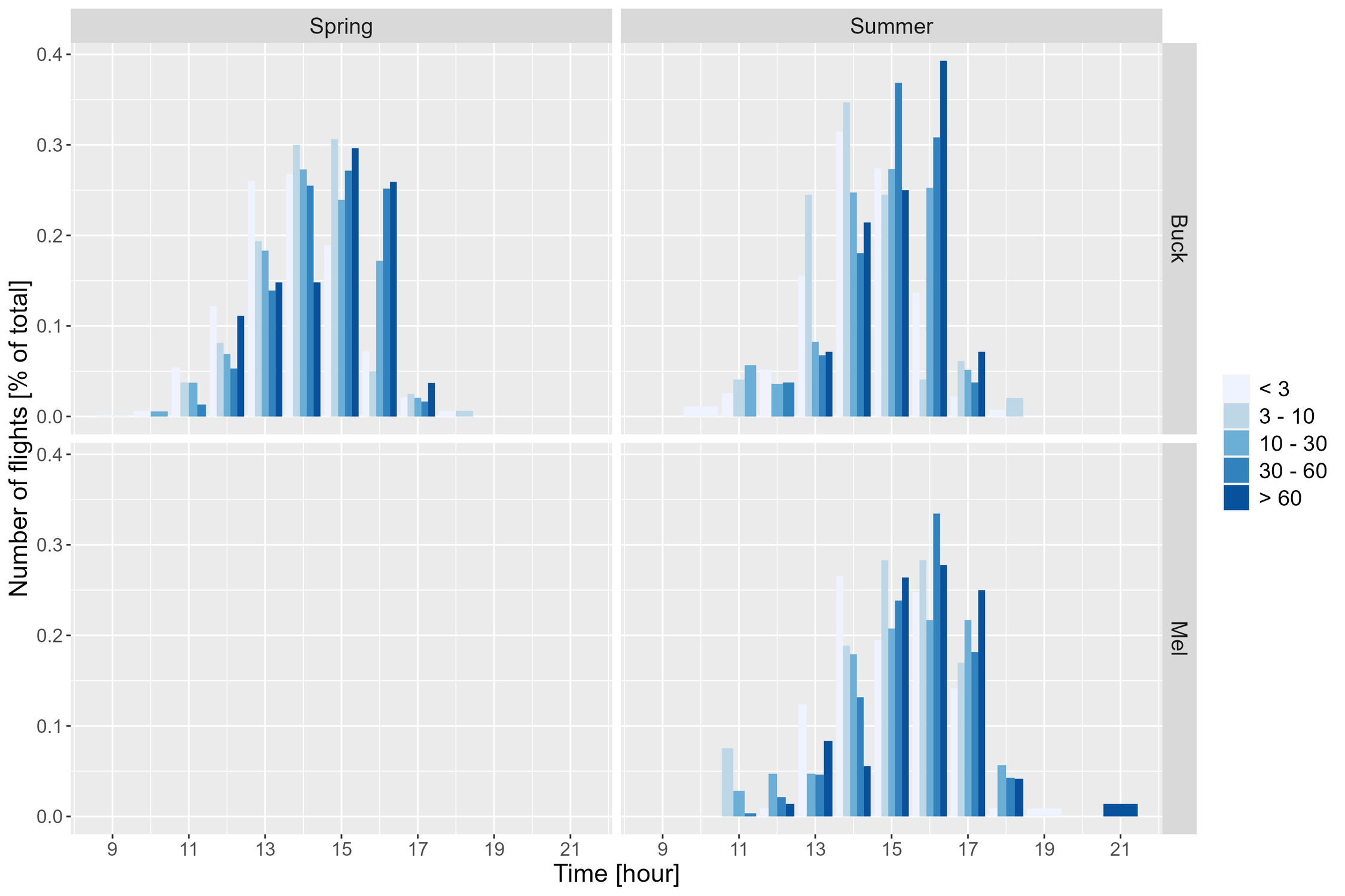

Supplement: S7 Fig — Relative numbers are given, thus the relation between the number of flights per hour to the total number of flights. The darker the colour palette, the longer the flight length. Longest flight lengths (mating flights) occurred during high activity times in the afternoon [Buck: hybrid Buckfast, Mel: Apis mellifera mellifera]. (TIF) [file pone.0308831.s007.tif]

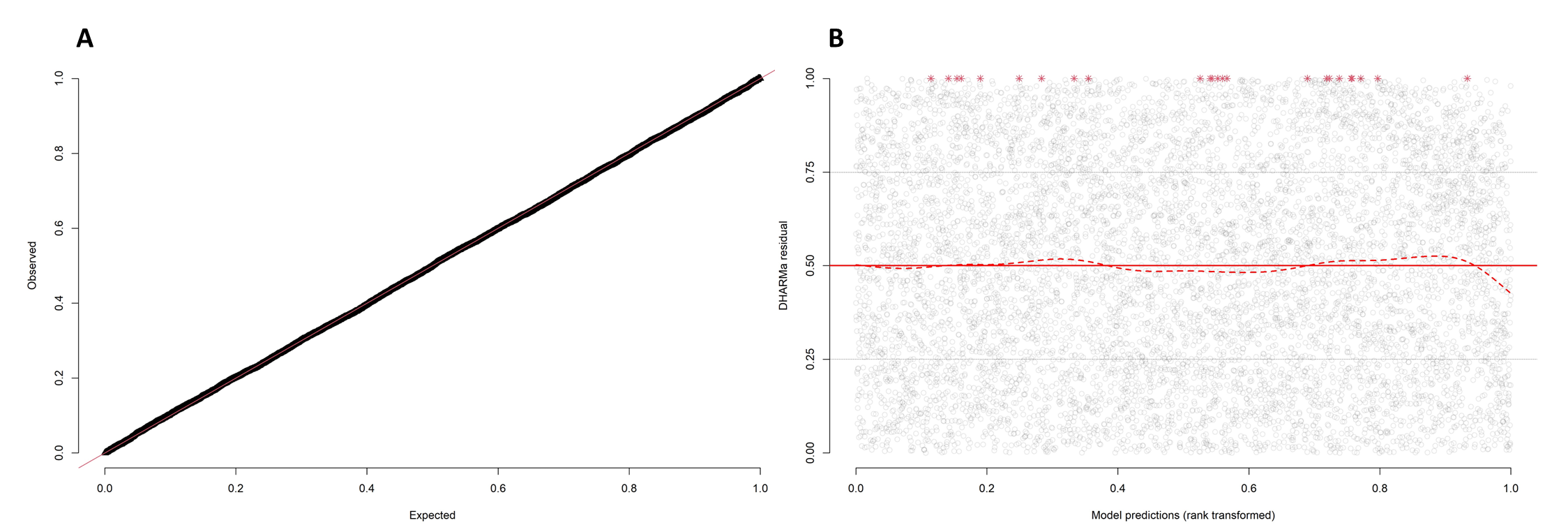

Supplement: S8 Fig — A) QQ residual plot and B) residual vs. predicted plot. A) and B) indicate an acceptable fit of the model. (TIF) [file pone.0308831.s008.tif]

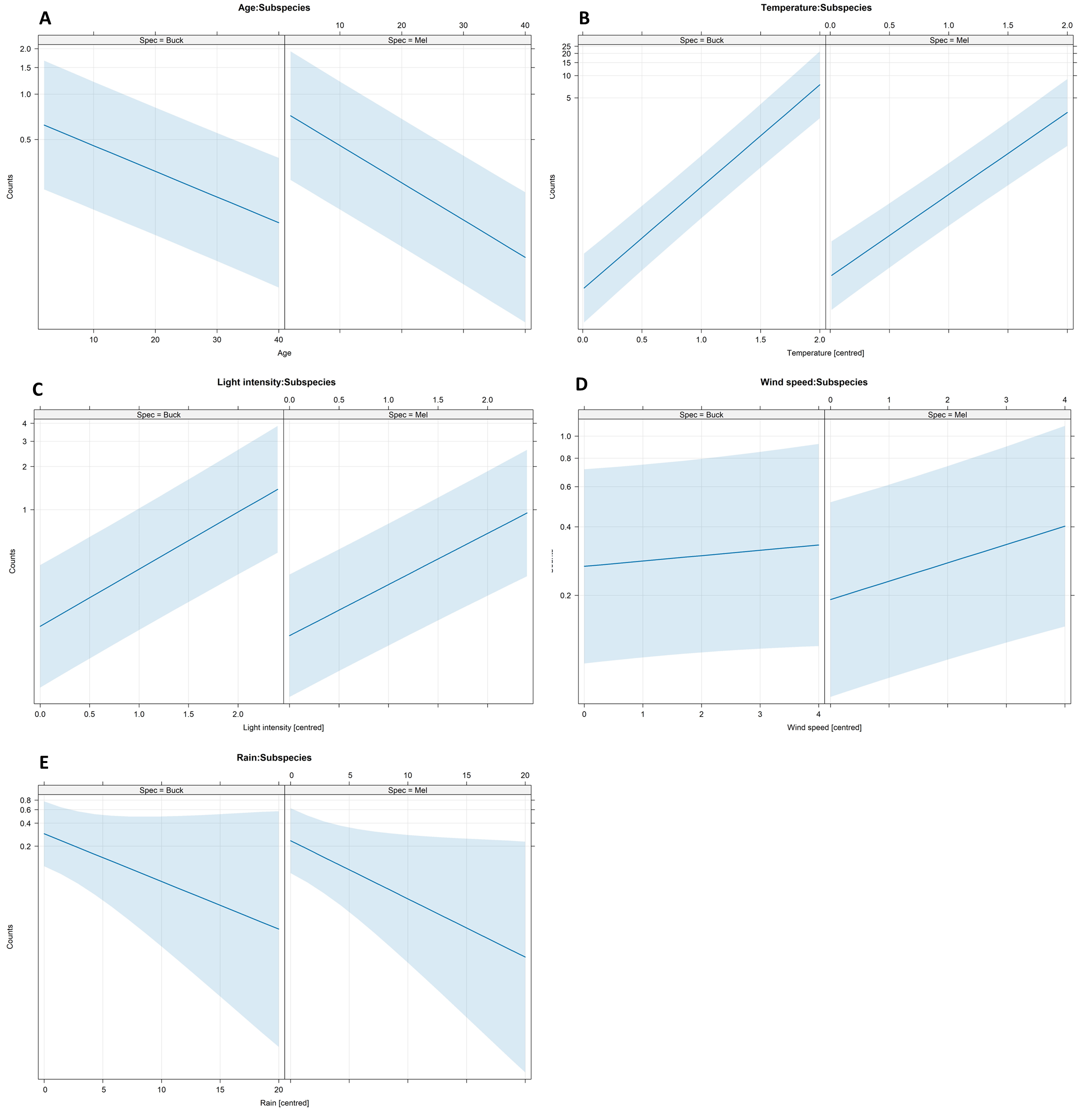

Supplement: S9 Fig — The effect of A) age, B) temperature, C) light intensity, D) wind speed and E) rain in interaction with subspecies on the number of hourly registrations for Buck and Mel worker bees. Significant differences were found in A) and B) [Buck: hybrid Buckfast, Mel: Apis mellifera mellifera]. (TIF) [file pone.0308831.s009.tif]

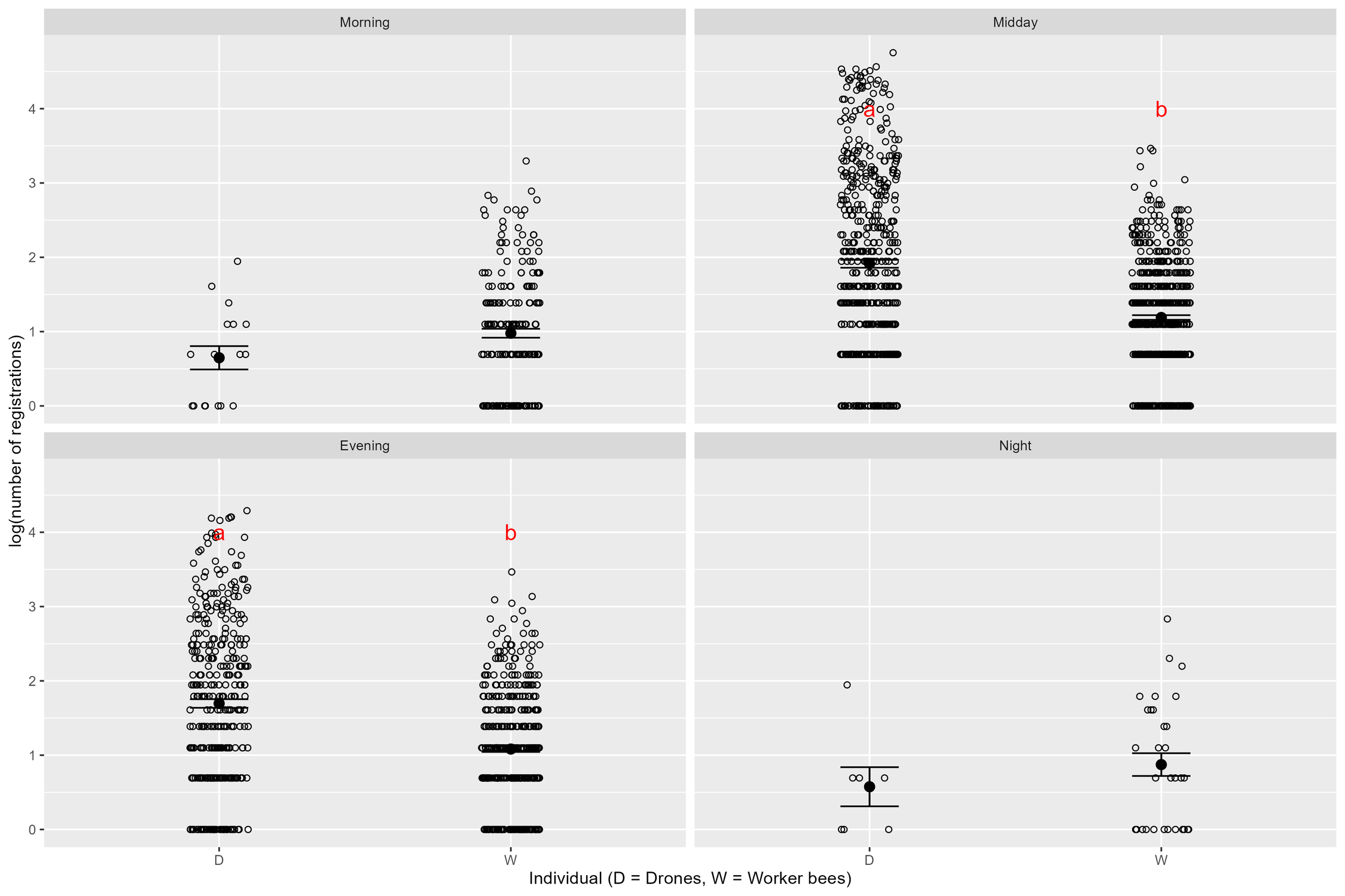

Supplement: S10 Fig — This figure shows the visualisation of the post hoc test of the ANOVA. The filled points display the mean, and the error bars show the standard error. The red letters indicate significant differences. Drones showed significantly more registrations during MIDDAY and EVENING than worker bees. (TIF) [file pone.0308831.s010.tif]

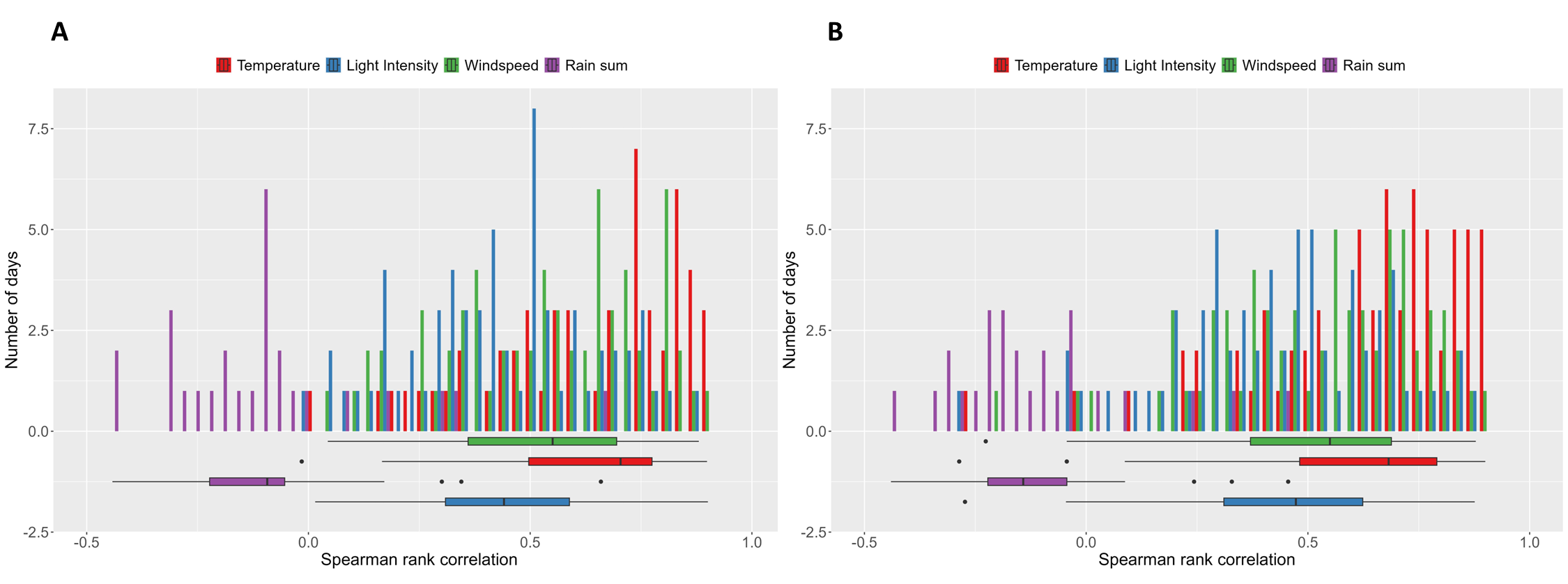

Supplement: S11 Fig — Daily spearman’s rank correlation of the number of registrations of A) Buck and B) Mel against temperature in red, light intensity in blue, wind speed in green and rain sum in purple. The boxplots at the bottom show the median (line), interquartile range (box) and the top and bottom one percentile (whiskers) for each weather parameter. Outliers are marked with black points [Buck: hybrid Buckfast, Mel: Apis mellifera mellifera]. (TIF) [file pone.0308831.s011.tif]

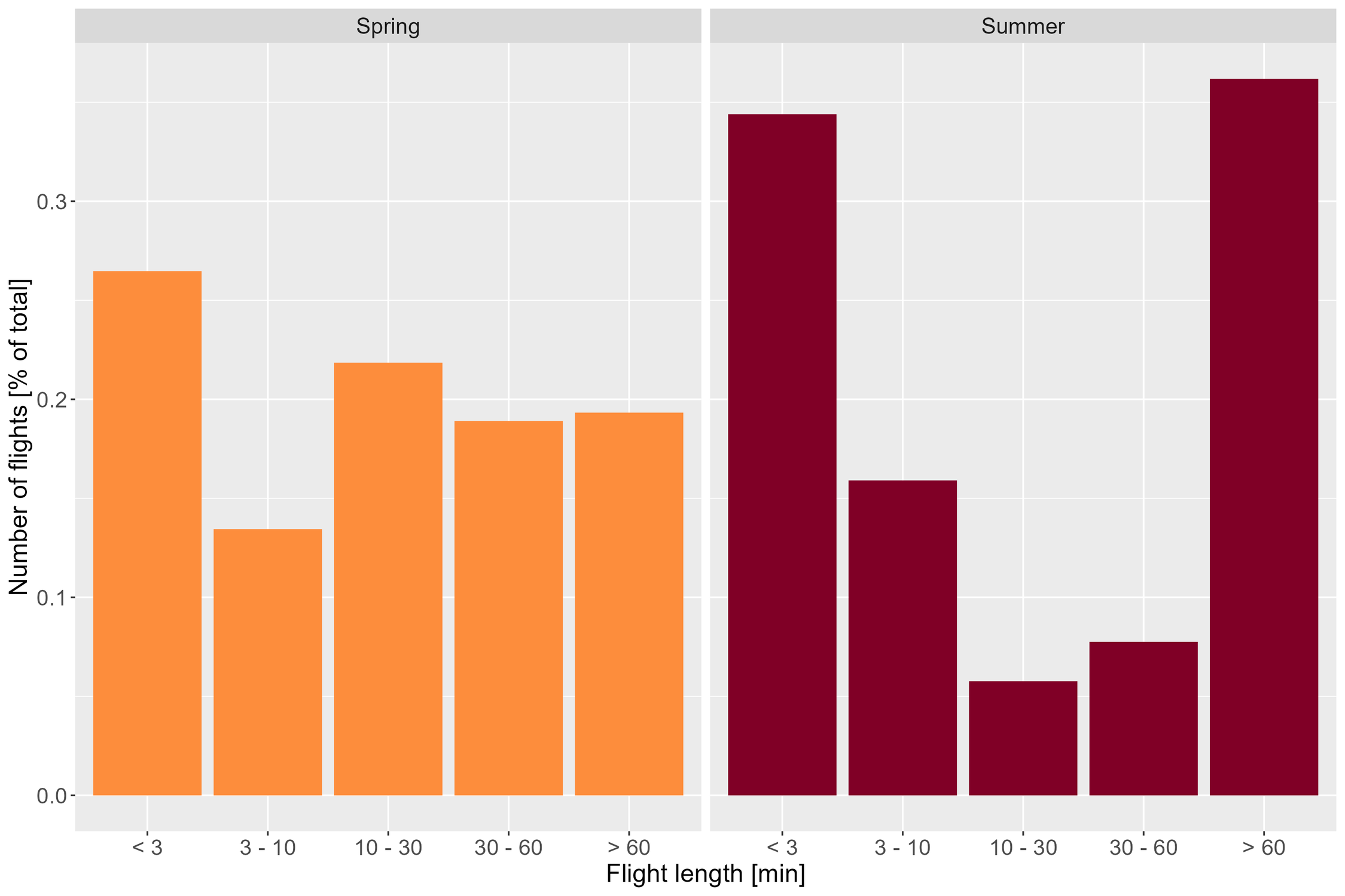

Supplement: S12 Fig — Relative numbers are given, thus the relation between the number of flights in a length category to the total number of flights. In summer, relatively more longer flights were performed by worker bees. (TIF) [file pone.0308831.s012.tif]
