# Supplementary material for: Ecological comparison of native (Apis mellifera mellifera) and hybrid (Buckfast) honeybee drones in southwestern Sweden indicates local adaptation
Source: PLoS One. 2024 Aug 13;19(8):e0308831. doi: 10.1371/journal.pone.0308831 (PMC11321565; doi:10.1371/journal.pone.0308831)
Supplement: S1 Table — Buck: hybrid Buckfast; Mel: Apis mellifera mellifera. (DOCX) [file pone.0308831.s013.docx]

| Subspecies | Breeder | Origin | Year |
| --- | --- | --- | --- |
| *Buck* | OAG | Gislaved, Jönköpings county | 2019 |
| *Mel* | Hammerdal | Hammerdal, Jämtlands county | 2021 |
| *Buck* | ASBgul | Skåne county, south | 2019 |
| *Mel* | MSB | Bygdeträsk, Västerbotten county | 2019 |
