# Supplementary material for: Ecological comparison of native (Apis mellifera mellifera) and hybrid (Buckfast) honeybee drones in southwestern Sweden indicates local adaptation
Source: PLoS One. 2024 Aug 13;19(8):e0308831. doi: 10.1371/journal.pone.0308831 (PMC11321565; doi:10.1371/journal.pone.0308831)
Supplement: S2 Table — Buck: hybrid Buckfast; Mel: Apis mellifera mellifera. (DOCX) [file pone.0308831.s014.docx]

|  | Drones | | Worker bees | |
| --- | --- | --- | --- | --- |
| Colony | **Spring** | **Summer** | **Spring** | **Summer** |
| OAG *Buck* | 28 | 30 | 20 | 20 |
| Hammerdal *Mel* | 0 | 30 | 20 | 20 |
| ASBgul *Buck* | 16 | 29 | 20 | 19 |
| MSB *Mel* | 0 | 30 | 19 | 20 |
| Total | 44 | 119 | 79 | 79 |
