# Supplementary material for: Ecological comparison of native (Apis mellifera mellifera) and hybrid (Buckfast) honeybee drones in southwestern Sweden indicates local adaptation
Source: PLoS One. 2024 Aug 13;19(8):e0308831. doi: 10.1371/journal.pone.0308831 (PMC11321565; doi:10.1371/journal.pone.0308831)
Supplement: S3 Table — Temp, Temperature (°C); Light, light intensity (PAR); Wind, wind speed (km/h); Rain sum (mm). Humidity was not analysed due to mainly high constant values (S1D Fig). (DOCX) [file pone.0308831.s015.docx]

| Month | Temp  minimum | Temp  average | Temp  maximum | Light average | Light maximum | Wind average | Wind maximum | Rain sum |
| --- | --- | --- | --- | --- | --- | --- | --- | --- |
| May | -3.6 | 11.0 | 22.6 | 88.4 | 360 | 5.8 | 31.7 | 58.5 |
| June | 4.3 | 16.6 | 33.3 | 108.8 | 376.6 | 4.2 | 27.4 | 29 |
| July | 4.6 | 17.7 | 34.8 | 97.7 | 372.7 | 3.8 | 23.8 | 52.3 |
| August | 3.0 | 17.3 | 33.0 | 82.2 | 342 | 2.8 | 23.8 | 96.3 |
