# Supplementary material for: Ecological comparison of native (Apis mellifera mellifera) and hybrid (Buckfast) honeybee drones in southwestern Sweden indicates local adaptation
Source: PLoS One. 2024 Aug 13;19(8):e0308831. doi: 10.1371/journal.pone.0308831 (PMC11321565; doi:10.1371/journal.pone.0308831)
Supplement: S4 Table — (DOCX) [file pone.0308831.s016.docx]

|  | Temperature | Light Intensity | Wind Speed | Rain |
| --- | --- | --- | --- | --- |
| Temperature | 1 | 0.454 | 0.286 | -0.004 |
| Light Intensity | 0.454 | 1 | 0.334 | -0.048 |
| Wind Speed | 0.286 | 0.334 | 1 | 0.028 |
| Rain | -0.004 | -0.048 | 0.028 | 1 |
