# Supplementary material for: Ecological comparison of native (Apis mellifera mellifera) and hybrid (Buckfast) honeybee drones in southwestern Sweden indicates local adaptation
Source: PLoS One. 2024 Aug 13;19(8):e0308831. doi: 10.1371/journal.pone.0308831 (PMC11321565; doi:10.1371/journal.pone.0308831)
Supplement: S5 Table — All model structures contained the same random effects and zero inflation formula. [A, Age; T, Temperature; L, Light intensity; W, Wind speed; R, Rain; S, Subspecies; D, Time interval]. (DOCX) [file pone.0308831.s017.docx]

| Model | df | AIC |
| --- | --- | --- |
| A + T + L + W + R + S + D + S:A + S:T + S:W + S:R + T:L | 20 | 11702.27 |
| A + T + L + W + R + S + D + S:A + S:T + S:W + S:R | 19 | 11788.58 |
| A + T + L + W + S + D + S:A + S:T + S:W + T:L | 18 | 11698.52 |
| A + T + L + W + S + D + S:A + S:T + S:W | 17 | 11786.41 |
