# Supplementary material for: Ecological comparison of native (Apis mellifera mellifera) and hybrid (Buckfast) honeybee drones in southwestern Sweden indicates local adaptation
Source: PLoS One. 2024 Aug 13;19(8):e0308831. doi: 10.1371/journal.pone.0308831 (PMC11321565; doi:10.1371/journal.pone.0308831)
Supplement: S6 Table — Significance codes: p < 0.001 = ***, p < 0.01 = **, p < 0.05 = *, p < 0.1 =. [Buck: hybrid Buckfast, Mel: Apis mellifera mellifera]. (DOCX) [file pone.0308831.s018.docx]

| Explanatory variable | Chisq | Df | Pr(>Chisq) |
| --- | --- | --- | --- |
| Age | 567.0717 | 1 | < 0.001 *** |
| Temperature | 377.6807 | 1 | < 0.001 *** |
| PAR | 200.0026 | 1 | < 0.001 *** |
| Wind | 0.1717 | 1 | 0.678 |
| Time interval | 310.2745 | 4 | < 0.001*** |
| Subspecies | 0.8983 | 1 | 0.343 |
| Temperature:PAR | 90.6719 | 1 | < 0.001*** |
| Subspecies:Age | 11.2732 | 4 | < 0.001 *** |
| Subspecies:Temperature | 2.8910 | 4 | 0.089 . |
| Subspecies:PAR | 1.4061 | 4 | 0.236 |
| Subspecies:Wind | 2.3341 | 4 | 0.126 |
