# Supplementary material for: Ecological comparison of native (Apis mellifera mellifera) and hybrid (Buckfast) honeybee drones in southwestern Sweden indicates local adaptation
Source: PLoS One. 2024 Aug 13;19(8):e0308831. doi: 10.1371/journal.pone.0308831 (PMC11321565; doi:10.1371/journal.pone.0308831)
Supplement: S7 Table — The 95% Confidence Intervals (CI) were used to detect significant differences between both subspecies. A significant difference in age was found [Buck: hybrid Buckfast, Mel: Apis mellifera mellifera]. (DOCX) [file pone.0308831.s019.docx]

| Parameter | Log-Mean | SE | 95 % CI | z | p |
| --- | --- | --- | --- | --- | --- |
| Age | -0.05 | 2.44e-03 | [-0.06, -0.05] | -21.00 | < 0.001 |
| Temperature | 4.67 | 0.28 | [4.12, 5.22] | 16.67 | < 0.001 |
| PAR | 2.72 | 0.20 | [2.32, 3.11] | 13.55 | < 0.001 |
| Wind | -0.02 | 0.05 | [-0.12, 0.07] | -0.52 | 0.6 |
| Time interval [Morning] | -8.20 | 0.68 | [-9.53, -6.87] | -12.11 | < 0.001 |
| Time interval [Midday] | -4.42 | 0.63 | [-5.66, -3.19] | -7.05 | < 0.001 |
| Time interval [Evening] | -4.35 | 0.63 | [-5.58, -3.12] | -6.92 | < 0.001 |
| Time interval [Night] | -7.03 | 0.70 | [-8.41, -5.65] | -9.99 | < 0.001 |
| Temperature x PAR | -1.35 | 0.14 | [-1.63, -1.08] | -9.52 | < 0.001 |
| Spec [Mel] | 0.70 | 0.53 | [-0.35, 1.74] | 1.31 | 0.190 |
| Spec [Mel] x Age | 0.01 | 3.83e-03 | [0.01, 0.02] | 3.36 | < 0.001 |
| Spec [Mel] x Temperature | -0.42 | 0.25 | [-0.90, 0.06] | -1.70 | 0.089 |
| Spec [Mel] x PAR | -0.12 | 0.11 | [-0.33, 0.08] | -1.19 | 0.236 |
| Spec [Mel] x Wind | 0.12 | 0.08 | [-0.03, 0.28] | 1.53 | 0.127 |
