# Supplementary material for: Ecological comparison of native (Apis mellifera mellifera) and hybrid (Buckfast) honeybee drones in southwestern Sweden indicates local adaptation
Source: PLoS One. 2024 Aug 13;19(8):e0308831. doi: 10.1371/journal.pone.0308831 (PMC11321565; doi:10.1371/journal.pone.0308831)
Supplement: S8 Table — Buck: hybrid Buckfast; Mel: Apis mellifera mellifera [Mdn: Median, IQR = Interquartile range]. (DOCX) [file pone.0308831.s020.docx]

|  | Count *Buck* | Count *Mel* |
| --- | --- | --- |
| Temperature | Mdn = 0.616, IQR = 0.170 | Mdn = 0.568, IQR = 0.156 |
| Light Intensity | Mdn = 0.331, IQR = 0.205 | Mdn = 0.228, IQR = 0.201 |
| Wind speed | Mdn = 0.499, IQR = 0.272 | Mdn = 0.495, IQR = 0.315 |
| Rain | Mdn = -0.109, IQR = 0.153 | Mdn = -0.125, IQR = 0.055 |
