# Supplementary material for: Ecological comparison of native (Apis mellifera mellifera) and hybrid (Buckfast) honeybee drones in southwestern Sweden indicates local adaptation
Source: PLoS One. 2024 Aug 13;19(8):e0308831. doi: 10.1371/journal.pone.0308831 (PMC11321565; doi:10.1371/journal.pone.0308831)
Supplement: S10 Table — All model structures contained the same random effects and zero inflation formula. [A, Age; T, Temperature; L, Light intensity; W, Wind speed; R, Rain; S, Subspecies; D, Time interval]. (DOCX) [file pone.0308831.s022.docx]

| Model | df | AIC |
| --- | --- | --- |
| A + T + L + R + W + D + S + S:A + S:T + S:L + S:R + S:W + T:L | 20 | 10488.61 |
| A + T + L + W + D + S + S:A + S:T + S:L + S:W + T:L | 18 | 10493.61 |
| A + T + L + W + D + S + S:A + S:T + S:L + S:W | 17 | 10650.97 |
| A + T + L + D + S + S:A + S:T + S:L + T:L | 16 | 10504.05 |
| A + T + L + D + S + S:A + S:T + S:L | 15 | 10669.66 |
