# Supplementary material for: Ecological comparison of native (Apis mellifera mellifera) and hybrid (Buckfast) honeybee drones in southwestern Sweden indicates local adaptation
Source: PLoS One. 2024 Aug 13;19(8):e0308831. doi: 10.1371/journal.pone.0308831 (PMC11321565; doi:10.1371/journal.pone.0308831)
Supplement: S11 Table — Significance codes: p < 0.001 = ***, p < 0.01 = **, p < 0.05 = *, p < 0.1 = . (DOCX) [file pone.0308831.s023.docx]

| Explanatory variable | Chisq | Df | Pr(>Chisq) |
| --- | --- | --- | --- |
| Age | 250.232 | 1 | < 0.001 *** |
| Temperature | 145.412 | 1 | < 0.001 *** |
| PAR | 34.422 | 1 | < 0.001 *** |
| Wind | 10.288 | 1 | 0.001 ** |
| Rain | 6.775 | 1 | 0.009 ** |
| Time interval | 167.991 | 4 | < 0.001 *** |
| Subspecies | 0.253 | 1 | 0.615 |
| Temperature:PAR | 143.702 | 1 | < 0.001 *** |
| Subspecies:Age | 10.386 | 4 | 0.001 ** |
| Subspecies:Temperature | 6.469 | 4 | 0.011 * |
| Subspecies:PAR | 0.973 | 4 | 0.324 |
| Subspecies:Wind | 3.089 | 4 | 0.078 |
| Subspecies:Rain | 0.067 | 4 | 0.796 |
