# Supplementary material for: Ecological comparison of native (Apis mellifera mellifera) and hybrid (Buckfast) honeybee drones in southwestern Sweden indicates local adaptation
Source: PLoS One. 2024 Aug 13;19(8):e0308831. doi: 10.1371/journal.pone.0308831 (PMC11321565; doi:10.1371/journal.pone.0308831)
Supplement: S12 Table — The 95% Confidence Intervals (CI) were used to detect significant differences between both subspecies. Significant differences in age and temperature were found [Mel: Apis mellifera mellifera]. (DOCX) [file pone.0308831.s024.docx]

| Parameter | Log-Mean | SE | 95 % CI | z | p |
| --- | --- | --- | --- | --- | --- |
| Age | -0.04 | 4.06e-03 | [-0.05, -0.03] | -9.66 | < 0.001 |
| Temperature | 4.38 | 0.27 | [3.84, 4.92] | 15.98 | < 0.001 |
| PAR | 2.67 | 0.21 | [2.26, 3.08] | 12.72 | < 0.001 |
| Wind | 0.05 | 0.06 | [-0.05, 0.16] | 0.98 | 0.327 |
| Rain | -0.14 | 0.09 | [-0.31, 0.03] | -1.64 | 0.102 |
| Time interval [Morning] | -4.90 | 0.59 | [-6.06, -3.75] | -8.30 | < 0.001 |
| Time interval [Midday] | -4.47 | 0.60 | [-5.64, -3.29] | -7.44 | < 0.001 |
| Time interval [Evening] | -4.65 | 0.60 | [-5.82, -3.48] | -7.78 | < 0.001 |
| Time interval [Night] | -6.25 | 0.60 | [-7.42, -5.08] | -10.46 | < 0.001 |
| Temperature:PAR | -1.80 | 0.15 | [-2.10, -1.51] | -11.99 | < 0.001 |
| Spec [Mel] | 0.74 | 0.75 | [-0.73, 2.21] | 0.98 | 0.325 |
| Spec [Mel] x Age | -0.02 | 5.49e-03 | [-0.03, -0.01] | -3.22 | 0.001 |
| Spec [Mel] x Temperature | -0.63 | 0.25 | [-1.11, -0.14] | -2.54 | 0.011 |
| Spec [Mel] x PAR | -0.10 | 0.10 | [-0.29, 0.09] | -0.99 | 0.324 |
| Spec [Mel] x Wind | 0.13 | 0.07 | [-0.02, 0.28] | 1.76 | 0.079 |
| Spec [Mel] x Rain | -0.03 | 0.12 | [-0.27, 0.21] | -0.26 | 0.796 |
