# Supplementary material for: Ecological comparison of native (Apis mellifera mellifera) and hybrid (Buckfast) honeybee drones in southwestern Sweden indicates local adaptation
Source: PLoS One. 2024 Aug 13;19(8):e0308831. doi: 10.1371/journal.pone.0308831 (PMC11321565; doi:10.1371/journal.pone.0308831)
Supplement: S13 Table — Buck: hybrid Buckfast, Mel: Apis mellifera mellifera [Mdn: Median, IQR = Interquartile range]. (DOCX) [file pone.0308831.s025.docx]

|  | Counts *Buck* | Counts *Mel* |
| --- | --- | --- |
| Temperature | Mdn = 0.682, IQR = 0.309 | Mdn = 0.703, IQR = 0.278 |
| Light Intensity | Mdn = 0.473, IQR = 0.313 | Mdn = 0.441, IQR = 0.279 |
| Wind speed | Mdn = 0.549, IQR = 0.317 | Mdn = 0.551, IQR = 0.335 |
| Rain | Mdn = -0.142, IQR = 0.178 | Mdn = -0.093, IQR = 0.169 |
